# Homology-Based Modeling of Universal Stress Protein from *Listeria innocua* Up-Regulated under Acid Stress Conditions

**DOI:** 10.3389/fmicb.2016.01998

**Published:** 2016-12-20

**Authors:** Patrizio Tremonte, Mariantonietta Succi, Raffaele Coppola, Elena Sorrentino, Luca Tipaldi, Gianluca Picariello, Gianfranco Pannella, Franca Fraternali

**Affiliations:** ^1^Department of Agricultural, Environmental and Food Sciences (DiAAA), University of MoliseCampobasso, Italy; ^2^Institute of Food Science, National Research Council (ISA-CNR)Avellino, Italy; ^3^Randall Division of Cellular and Molecular Biophysics, New Hunt's House King's CollegeLondon, UK

**Keywords:** universal stress protein, acid stress, *Listeria*, exponential growth phase, homology modeling, 2-D Electrophoresis, ATP-binding motif

## Abstract

An Universal Stress Protein (USP) expressed under acid stress condition by *Listeria innocua* ATCC 33090 was investigated. The USP was up-regulated not only in the stationary phase but also during the exponential growth phase. The three dimensional (3D) structure of USP was predicted using a combined proteomic and bioinformatics approach. Phylogenetic analysis showed that the USP from *Listeria* detected in our study was distant from the USPs of other bacteria (such as *Pseudomonas* spp., *Escherichia coli, Salmonella* spp.) and clustered in a separate and heterogeneous class including several USPs from *Listeria* spp. and *Lactobacillus* spp. An important information on the studied USP was obtained from the 3D-structure established through the homology modeling procedure. In detail, the Model_USP-691 suggested that the investigated USP had a homo-tetrameric quaternary structure. Each monomer presented an architecture analogous to the Rossmann-like α/β-fold with five parallel β-strands, and four α-helices. The analysis of monomer-monomer interfaces and quality of the structure alignments confirmed the model reliability. In fact, the structurally and sequentially conserved hydrophobic residues of the β-strand 5 (in particular the residues V^146^ and V^148^) were involved in the inter-chains contact. Moreover, the highly conserved residues I^139^ and H^141^ in the region α4 were involved in the dimer association and functioned as hot spots into monomer–monomer interface assembly. The hypothetical assembly of dimers was also supported by the large interface area and by the negative value of solvation free energy gain upon interface interaction. Finally, the structurally conserved ATP-binding motif G-2X-G-9X-G(S/T-N) suggested for a putative role of ATP in stabilizing the tetrameric assembly of the USP. Therefore, the results obtained from a multiple approach, consisting in the application of kinetic, proteomic, phylogenetic and modeling analyses, suggest that *Listeria* USP could be considered a new type of ATP-binding USP involved in the response to acid stress condition during the exponential growth phase.

## Introduction

*Listeria innocua* is regarded as a non-pathogenic indicator for the presence of *Listeria monocytogenes* in foods (Rosimin et al., 2016). Numerous ecological and genomic comparative studies highlighted a high similarity between the two species (Glaser et al., 2001; Girardin et al., 2005; den Bakker et al., 2010). Similarly to *L. innocua*, pathogenic *L. monocytogenes* is frequently found in various foodstuffs (Kovacevic et al., 2012; Jami et al., 2014; Ebner et al., 2015; Melo et al., 2015), especially those characterized by pH values higher than 4.4 (CAC, 2009). In the food industry a number of strategies are used to inhibit the growth of undesirable microorganisms, like technological processing (Tremonte et al., 2014), addition of natural substances (Tipaldi et al., 2011; Tremonte et al., 2016), protective microbial cultures (Sorrentino et al., 2013) as well as organic acids (Davidson et al., 2013). Unfortunately, in the case of several food types, sub-lethal pH values may induce resistance mechanisms to acid stress, which make the cells more resistant to severe acid conditions (Gandhi and Chikindas, 2007).

Acid stress response in *L. monocytogenes* has been the subject of several investigations, which documented the induction of a number of molecular mechanisms involving the F_1_F_0_-ATPase complex, the arginine deaminase (ADI) and the glutamate decarboxylase (GAD) pathways (Cotter et al., 2001; Ryan et al., 2009; Karatzas et al., 2012). On the other hand, little information is available on the role of Universal Stress Proteins (USPs) in the stress response of *Listeria* spp., although the expression of USPs was already studied in numerous other microorganisms (Tkaczuk et al., 2013).

USPs are cytoplasmic proteins found in Bacteria and Archea, as well as in fungi and plants. Their production is stimulated by several types of environmental stress or by specific physiological cell conditions (Kvint et al., 2003). Recently, a genomic approach demonstrated the involvement of *usp* encoding genes in the survival of *L. monocytogenes* in acid or oxidative stress conditions (Seifart Gomes et al., 2011), but the key role of USPs into cellular mechanisms remains generally unclear. To understand the molecular basis of possible USPs functions, the knowledge of their three-dimensional (3D) structures is essential. Amongst the ca. 110,000 structure deposited in the Protein Databank (PDB) only a few USPs structures have been determined so far. Indeed, the 3D-structures for USPs of *Listeria* are not available as of today. This gap may be filled by bioinformatics approaches such as homology modeling, on the condition that the sequence identity with known related structures is above 30% (Marti-Renom et al., 2000). Homology or “comparative” modeling, use an experimentally determined structure of a related protein as a template to model the structure of a target protein, and is the method of choice in the case of close sequence relationship (Petry and Honing, 2005). This approach is based on the observation that evolutionary and functionally related proteins generally share similar 3D structures. In this work, we used complementary proteomic and bioinformatics approaches in order to characterize USP proteins up-regulated in *L. innocua* ATCC 33090 under acid stress conditions and to predict *in silico* their 3D structure by referring to available template homologs in PDB.

## Materials and methods

### Bacterial strain and growth condition

*L. innocua* ATCC 33090, obtained from the Leibniz Institute DSMZ-German Collection of Microorganisms and Cell cultures, was revitalized in Brain Heart Infusion broth (BHI; Oxoid, Milan, Italy) at 37°C and then stored in cryovials (Pro-Lab Diagnostics, Richmond Hill, Canada) at −80°C. Prior to use, cells were propagated twice in the same medium and incubation conditions, and collected in the middle of exponential phase. The growth was assessed for 30 h in 500 mL of BHI (conventional condition, batch C) and in BHI adjusted at pH 4.5 (acid stress condition, batch AS). Moreover, a further trial was performed using pre-acid-adapted cells. For this purpose, cells cultured under acid stress condition were collected in the middle of exponential phase and inoculated in BHI (batch pa-C) and in BHI at pH 4.5 (batch pa-AS). In all cases an initial inoculum of about 10^7^ CFU/mL was used.

Plate counts were performed in BHI agar at different intervals and the growth kinetic parameters were estimated with the D-model of Baranyi and Roberts (1994) using the excel add-in DMFit v.3 (Baranyi and Le Marc, 1996). Three independent experiments were performed and the results were reported as average.

### Protein extraction

Cells of *L. innocua* ATCC 33090 cultivated as described above (batches C, AS, pa-C, and pa-AS) were collected by centrifugation (7500 rcf at 4°C for 15 min, Centrifuge Eppendorf, 5804R) in the middle of the exponential phase and during the stationary phase. Cells were washed three times with Tris-HCl (50 mM, pH 7.5), standardized at an OD_600_-value of 1.0 and re-suspended in a lysis buffer (Tris-HCl 50 mM, lysozyme 2 mg/mL, mutanolysin 50 U, protease inhibitor cocktail 1X, pH 7.5). Eight glass beads (Ø 0.4 mm) were added to each cellular suspension (140 μL), then suspensions were vortexed (3 min), incubated for 2 h at 37°C, vortexed (3 min), and sonicated for 5 min with an ultrasonic homogenizer (100 W power, 100% amplitude, 0.8 cycle; Labsonic M, Sartorius, Italy) using a probe of 0.5 mm diameter. After centrifugation at 17,500 rcf for 30 min at 4°C, the pellet was discarded and the supernatant (lysis buffer), containing the protein extract, was subjected to the Bradford-based protein assay kit (Bradford protein assay, Bio-Rad, Italy) to determine the protein concentration. Bovine serum albumin was used as a standard.

### Two-dimensional gel electrophoresis (2-DE)

Proteins of *L. innocua* ATCC 33090, cultivated as above (batches C, AS, pa-C, and pa-AS), were collected from the lysis buffer with methanol/chloroform according to the method described by Wessel and Flügge (1984). Isoelectrofocusing (IEF) was performed using precast immobilized pH gradient (IPG) 24 cm strips with a pI 4–7 linear gradient. IPG strips were passively rehydrated for 21 h with a buffer containing urea 8 M, CHAPS 2%, DTT 50 mM, 2% of ampholine 3/10, bromophenol blue 0.002%, and 800 μg of proteins. IEF was performed at 65,000 Vh using the Ettan IPGphor apparatus (GE Healthcare Bio). Prior to 2-DE, the strips were equilibrated for 25 min with 50mM Tris-HCl, pH 8.8, urea 6M, glycerol 30% (v/v), SDS 2% (w/v), bromophenol blue 0.002% (w/v) containing alternatively 65 mM of dithiothreitol (DTT) or 70 mM iodoacetamide. The SDS-PAGE separation was performed at constant current (18 mA/gel) and temperature (15°C) using the Ettan DALTsix Electrophoresis System apparatus (Amersham, Bio-sciences). Gels were stained for 2 h with the Bio-Safe colloidal Coomassie Blue G-250 (Bio-Rad) and digitalized using a GS-800 calibrated densitometer (Bio-Rad).

The 2-DE protein patterns were recorded as digitalized images using the Densitometer Calibrate GS–800 (Bio-Rad, Hercules, CA, USA). Spot detection, quantification, and analysis, were performed using the PDQuest™ 2-D gel analysis software, Version-8 (Bio-Rad, Hercules, CA, USA).

### Protein identification by mass spectrometry

Proteins with modified expression level were identified by matrix assisted laser desorption ionization-time of flight (MALDI-TOF) mass spectrometry (MS)-based peptide mass fingerprinting (PMF). Briefly, protein spots were manually excised, destained, and digested overnight at 37°C with 12 ng/mL proteomic grade trypsin (Promega, Madison, WI, USA). Afterwards, peptides were extracted in 1% formic acid/acetonitrile (1:1), vacuum dried, and analyzed using α-cyano-4-hydroxycinnamic acid (10 mg/mL in 50% acetonitrile/0.1 TFA) as the matrix. Mass spectra were obtained in the reflectron positive ion mode on a MALDI-TOF Voyager-DE™ PRO mass spectrometer (Applied Biosystems) equipped with a 337 nm N_2_ laser, acquiring at least 400 laser shots from each sample. A peptide mixture (Sigma-Aldrich Co.) was used as external standard. Proteins were identified with the MS-Fit Protein Prospector software (website: http://prospector.ucsf.edu), searching the Uniprot database (2015.3.5 vers.). C-carbamidomethylation was set as a fix modification, while pyroglutamic acid at N-terminal glutamine and methionine oxidation were variable modifications. Up to one trypsin missed cleavage was allowed and peptide mass tolerance was 40 ppm. Search was taxonomically limited to microorganisms and then refined, with taxonomical restriction to *Listeria* spp. Only protein hits with at least 15 matching peptides, MOWSE score higher than 10^8^, and coverage higher than 25% were considered as positive identifications. Protein identification was validated by a software-assisted comparison of experimental and expected Mw and pI, as inferred from the UniProtKB/Swis-Prot database through the TagIdent tool (http://web.expasy.org/tagident/).

### Target and template selection for USPs sequences alignment

**Figure 1:**
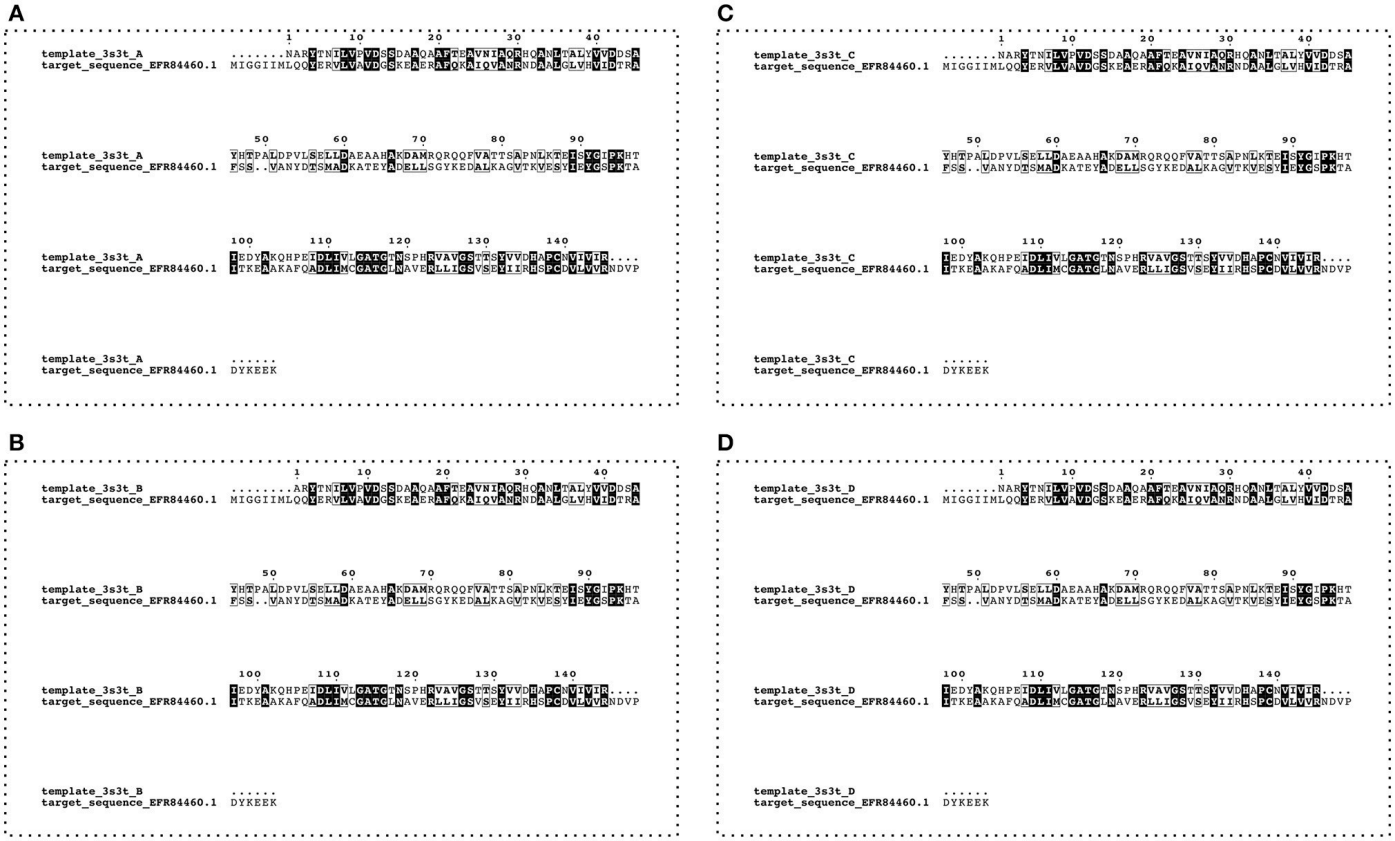
**Alignment between target (Uniprot accession number A0A0E1Y4Z4—***L. monocytogenes*** FSL F2-208) and template (four chains—A, B, C,** and **D**—from PDB code:3s3t—*Lb. plantarum*) USP sequences. Conserved residues are highlighted by the boxes.

Based on the mass spectrometry results, the FASTA sequence (160 aa) of the USP (Uniprot accession no. A0A0E1Y4Z4) from *L. monocytogenes* FSL F2-208 was used as target. Homolog sequences and the USP template sequence were searched in the non-redundant protein sequence database (NR) and Protein Data Bank (PDB) respectively, using the protein-protein Basic Local Alignment Search Tool (BLASTp). The USP structure (PDB ID: 3S3T) from *Lactobacillus plantarum* was chosen as template for the model building due to its protein sequence showing the highest alignment score (31% of identity and E-value of 1^−21^). In detail, the four homolog chains A, B, C, and D of the complete USP structure from *Lb. plantarum* were aligned with the target sequence of *Listeria* (Figure 1). Sequence alignments were performed with the PRALINE program server (Simossis and Heringa, 2005) and edited with Jalview (Waterhouse et al., 2009).

### USPs phylogenetic analysis

The USP target from *L. monocytogenes* FSL F2-208 and other 54 bacterial domains of USPs previously characterized in other studies were used in the phylogenetic analysis. For this purpose, a multiple sequence alignment was constructed using ClustalW algorithm with Gonnet substitution matrix, gap open penalty of 10, and gap extension penalty of 0.2 (Thompson et al., 1994). Phylogenetic tree calculation was performed with the Neighbor-Joining method (Saitou and Nei, 1987) using MEGA7 software (Kumar et al., 2016). The statistical significance of the phylogenetic tree was tested by using bootstrap analysis (Felsenstein, 1985), with each bootstrap value reflecting the confidence of each branch.

### Model building and validation

Comparative homology modeling (Sali and Blundell, 1993) was used to build a model of *L. innocua* USP by means of the MODELLER software (Marti-Renom et al., 2000; Webb and Sali, 2014) and generating 1000 models. The model with the lower Discrete Optimized Potential Energy (DOPE) score was submitted to a Procheck (Laskowski et al., 1993) analysis for a preliminary investigation of the model structure stereochemical quality. The model was thereafter refined by the use of the KoBaMIN web server, which consist applying a protein structure refinement protocol based on the minimization of a knowledge-based potential of mean force (Rodrigues et al., 2012). The final model was validated via the Procheck and QMEAN Server (Benkert et al., 2009). The QMEAN Z-score was used in the evaluation, providing an estimation of the absolute quality of a model by relating it to reference structures solved by X-ray crystallography (Benkert et al., 2011). The Root Mean Squared Deviation (RMSD) was calculated to evaluate the similarity between the 3D structure of the template and the model. PyMol software was used for the RMSD calculation and to generate model images.

### Interfaces analysis and structure alignments

The interfaces were explored using the Webservers PISA (Krissinel and Henrick, 2007) and POPSCOMP (Kleinjung and Fraternali, 2005). Moreover, the electrostatic potential distribution was calculated using the APBS (Adaptive Poisson-Boltzman Solver) and PDB2PQR software packages (Baker et al., 2001; Dolinsky et al., 2004, 2007) and mapped onto a molecular surface of protein model using the PyMol software. Additional template structures were searched with the DALI sever (Holm and Rosenström, 2010). The structural alignment program MUSTANG was used to perform multiple structural alignments (Konagurthu et al., 2006, 2010). Sequence alignments were displayed with ESPript (Robert and Gouet, 2014).

### Statistical analysis

Statistical analysis was performed on three independent experiments through ANOVA followed by the Tuckey's mulptiple comparison. For this purpose, the software GraphPad Prism version 6.0 was used.

## Results

### Microbial growth of *Listeria innocua* in acid condition

**Figure 2:**
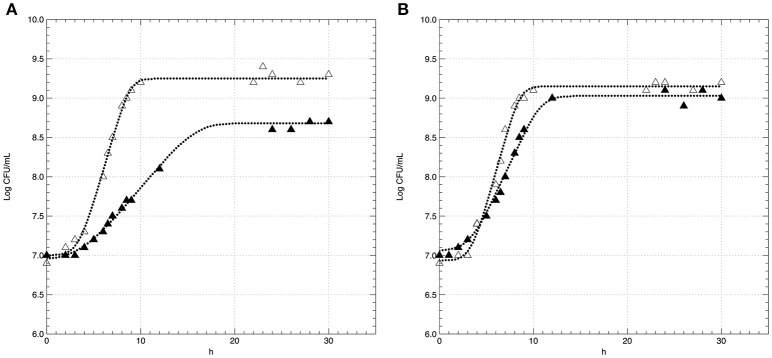
**Growth curves of ***L. innocua*** ATCC 33090 cultivated in conventional (Δ) and in acid stress (▲) conditions after non-pre-acid-adaptation (A)** or pre-acid-adaptation **(B)**. Symbols represents the experimental data, dotted curves represents the D-model.

The effect of conventional (C) and acid stress (AS) conditions on the growth kinetics of non-pre-acid-adapted cells of *L. innocua* are reported in Figure 2A. The figure also highlights the kinetic curves of pre-acid-adapted cells cultivated in conventional (pa-C) and in acid stress (pa-AS) conditions (Figure 2B). The results evidenced that the acid stress condition strongly affected the kinetic parameters (Table 1) and the effect was particularly noticeable considering the maximum specific growth rate (μ_max_). In fact, the non-pre-acid-adapted cells (Figure 2A) showed a lower growth rate when cultivated in acid condition, as evidenced by μ_max_-values three-fold lower in AS (μ_max_ of about 0.43 h^−1^) than that exhibited in C (μ_max_ of about 0.14 h^−1^). Moreover, non-pre-acid-adapted cells cultured in conventional conditions (C) reached the stationary phase (y_end_ ≈1.9^9^ CFU/mL) in about 12 h, whereas in acid stress conditions (AS) the maximum population (y_end_ ≈5.2^8^ CFU/mL) was reached in about 20 h (Figure 2A and Table 1). Conversely, low or no differences were detected between the batches pa-C and pa-AS considering all the kinetic parameters. In this case, the μ_max_ was about 0.42 h^−1^ for pa-C condition and about 0.30 h^−1^ for pa-AS. Moreover, no significant differences (*p* > 0.05) between pa-C and pa-AS were found in the final microbial levels (y_end_, Table 1).

**Table 1:** **Growth kinetic parameters of ***L. innocua*** ATCC 33090 cultivated in conventional and acid stress conditions after non-pre-acid-adapted or pre-acid-adaptation**.

	**μ_max_ (h^−1^)**	**λ (h)**	**y_in_ (Log CFU/mL)**	**y_end_ (Log CFU/mL)**	***R*^2^**
C	0.43 ± 0.03^a^	3.4 ± 0.1^a,b^	7.0 ± 0.1^a^	9.3 ± 0.1^a^	0.992
AS	0.14 ± 0.02^b^	3.7 ± 0.1^b^	6.9 ± 0.2^a^	8.7 ± 0.1^b^	0.994
pa-C	0.42 ± 0.02^a^	3.3 ± 0.1^a,b^	6.9 ± 0.2^a^	9.2 ± 0.1^a,c^	0.996
pa-AS	0.30 ± 0.02^c^	3.2 ± 0.3^a^	7.1 ± 0.1^a^	9.0 ± 0.1^c^	0.985

*Mean ± standard deviation of three independent experiments. Means in the same column with different superscript small letters are significantly different (p <0.05)*.

### Protein expression in exponential phase under acid stress conditions

**Figure 3:**
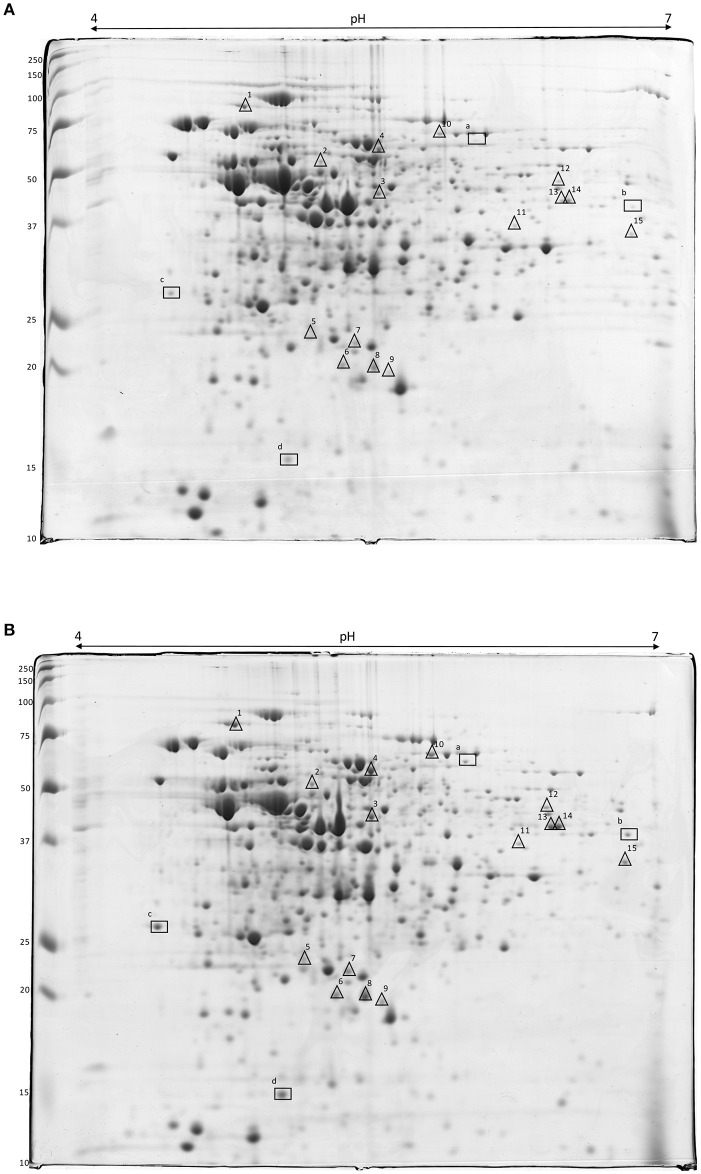
**2-DE gels of crude extract proteins of ***L. innocua*** ATCC 33090 cultivated in conventional (A)** and in acid stress **(B)** conditions. Symbols show the spot proteins strongly (Δ) or very strongly (□) up-regulated in acid stress condition.

Proteins were extracted from cells of *L. innocua* ATCC 33090 in the exponential growth phase and the proteome of cells cultivated in conventional (C, pa-C) and acid stress (AS, pa-AS) conditions was compared by 2-DE. More than 500 spots were detected in each gel apart from the cultural conditions. The image analysis highlighted significant differences between conventional and acidic stress conditions. In detail, an up-regulation of several spot proteins was detected in the proteome of cells cultivated in acid stress conditions (AS, pa-AS). In detail, after normalization over a number of protein spots with unmodified expression, densitometric analysis of the spot intensity evidenced 19 differentially expressed gene products at a threshold ≥2 (*p* < 0.05), which were selected for the MS-based identification (Figure 3 and Table 2).

**Table 2:** **Main proteins up-regulated in ***L. innocua*** ATCC 33090 cultivated in acid stress conditions**.

**Spot ID**	**Mr (Da)**	**pI**	**Accession no**.	**Protein name**	**Induction ratio**
a	53193	5.60	gi|489862543	Succinate-semialdehyde dehydrogenase	8.0
b	37800	5.91	H1G8Z4	NADP-dependent aryl-alcohol dehydrogenase	5.1
c	22600	4.38	Q92F64	General stress protein CTC	6.4
d	17555	5.00	A0A0E1Y4Z4	Universal stress protein	3.0
1	57300	4.70	Q929V0	60 kDa chaperonin, GroEL	2.4
2	48150	5.10	Q92BG1	Glutamate-1-semialdehyde 2,1-aminomutase 1	2.4
3	41140	5.29	Q71Z79	Acetylornithine aminotransferase	2.2
4	51080	5.29	B8DBU2	3-isopropylmalate dehydratase large subunit	2.2
5	21180	5.07	Q92AC4	Xanthine phosphoribosyltransferase	2.0
6	20330	5.17	B8DH16	Pyridoxal 5′-phosphate synthase subunit PdxT	2.0
7	21080	5.22	B8DG13	Ribosome-recycling factor	2.3
8	20300	5.27	A0AJW5	Peptide methionine sulfoxide reductase MsrA	2.7
9	20130	5.33	Q8Y7I9	S-ribosylhomocysteine lyase	2.1
10	55160	5.49	Q8Y5M1	UDP-N-acetylmuramoylalanine–D-glutamate ligase	2.8
11	34890	5.67	Q92BR0	Probable endonuclease 4	2.1
12	43510	5.75	Q8Y5N2	Quinolinate synthase A	2.8
13	39740	5.76	Q8Y4L8	Methionine import ATP-binding protein MetN 2	2.9
14	39700	5.77	P0DJL8	Alanine racemase	2.7
15	32050	5.90	C1KVV7	N-acetylmuramic acid 6-phosphate etherase	2.8

Four protein spots (a, b, c, and d) were highly up-regulated, with expression ratio ranging from 3 to 8, namely succinate-semialdehyde dehydrogenase, NADP-dependent aryl-alcohol dehydrogenase, general stress protein (CTC), and (USP). Using the MS-based approach, the USP was identified in the proteome of *L. innocua* (Uniprot No H1GE00) but the primary structure was identical to the USP from *L. monocytogenes* (Uniprot accession number A0A0E1Y4Z4), sharing each other 100% of structural homology (Table 2).

### USP expression as a function of growth phase and acid conditions

**Figure 4:**
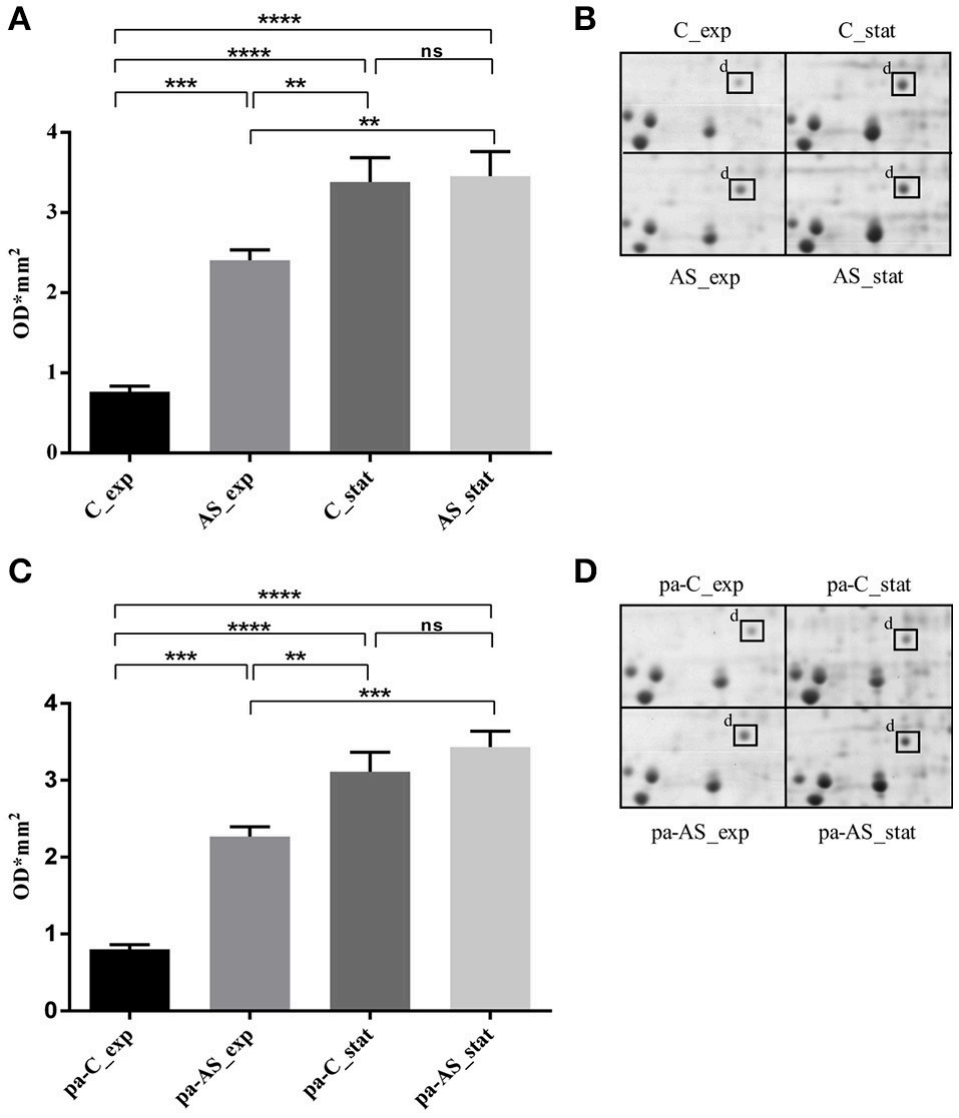
**Expression of ***L. innocua*** ATCC 33090 USP under physiological or acid stress condition represented as spot area revealed in non-pre-acid-adapted cells (A)** or in pre-acid-adapted cells **(C)** and as high magnification of 2-DE gels of the proteome from non-pre-acid-adapted cells **(B)** or in pre-acid-adapted cells **(D)**. Spots area values are the average of three replicate experiments and the error bars represent the standard deviation. The ^*^indicates that the difference is statistically significant as determined by one-way ANOVA followed by the Tuckey's multiple comparison test (^**^p < 0.05), (^***^p = 0.0001), (^****^p < 0.0001) while (*ns*) indicates that no statistical differences were detected.

A targeted 2-DE analysis was performed to investigate the expression of the USP in *L. innocua* in different conditions (Figure 4). The densitometric quantification of the protein spots evidenced that USP expression was significantly dependent on the growth phase, the cultivation in acidified medium and the pre-acid-adaptation conditions. The high magnification of 2-DE gels (Figure 4B) and the statistical analysis of spot area (Figure 4A) highlighted that when non-pre-acid-adapted cells were cultured in conventional conditions, the USP was up-regulated more than four-fold in the stationary phase (C_stat) compared to the exponential phase (C_exp). The cultivation in acid condition significantly affected the USP expression not only in the stationary phase (AS_stat) but also in the exponential phase (AS_exp). In fact, when non-pre-acid-adapted cells were cultured in acid conditions, the USP was already up-regulated in the exponential phase (AS_exp).

Similar results were obtained for pre-acid-adpted cells (Figures 4C,D). However, when cells were reinoculated in the conventional conditions (pa-C_exp), a down-regulation of the USP expression was observed during the exponential phase compared to that revealed during the same phase in acid condition.

### A functional model for the USP protein

**Figure 5:**
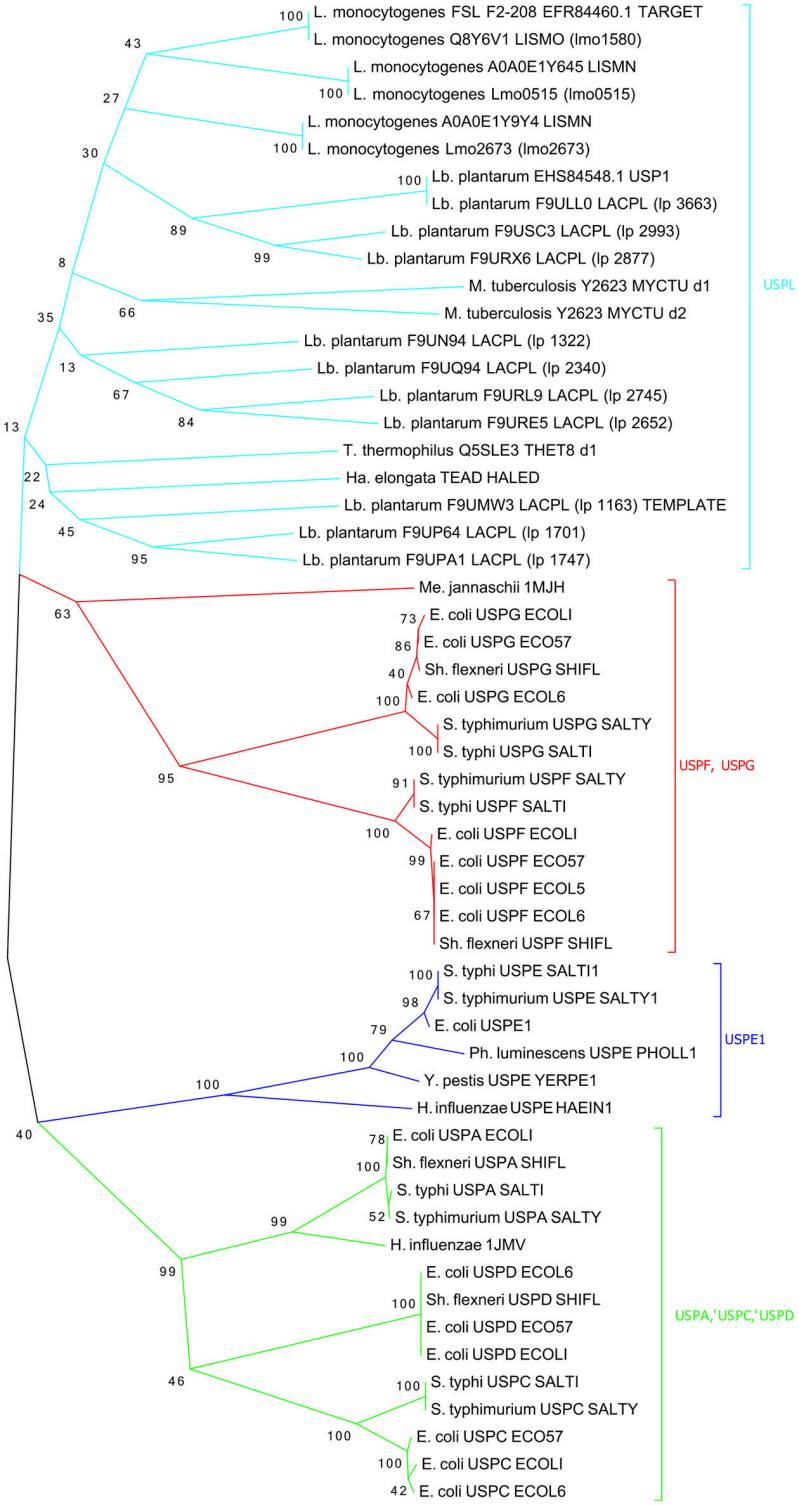
**Phylogenetic tree of the 55 most characterized USPs**. Bacterium genus are indicated: (L.), *Listeria*; (Lb.), *Lactobacillus*; (S.), *Salmonella*; (Sh.), *Shigella*; (Y.), *Yersinia*; (Ph.), *Photorhabdus*; (E.), *Escherichia*; (H.), *Haemophylus*; (Ha.), *Halomonas*; (T.), *Thermus*; (M.), *Mycobacterium*; (Me.), *Methanococcus*.

USPs proteins are highly conserved and all deemed important for the stress response. The FASTA sequence (160 aa) of USP from *L. monocytogenes* FSL F2-208 was obtained from the Uniprot database (Uniprot No A0A0E1Y4Z4). Similar sequences were searched into non-redundant protein sequences database using BLASTp. About 100 protein sequences, with 140–161 residues, showed a high sequence identity (56–100%) with a score ranging from 181 to 314 and a E-value from 9e^−55^ to 6e^−107^. The multi-sequences alignment (Figure S1) showed highly conserved regions especially between the residues 14 and 54 and between the residues 95 and 150. The conserved regions contain functionally relevant residues (Nachin et al., 2008). Moreover, phylogenetic analysis of the refined structural models of USPs could be exploited for further important functional information. To this purpose, a phylogenetic tree was constructed using 55 bacterial USP domain sequences and was divided into four clusters (Figure 5). In agreement with previous results (Nachin et al., 2008; Gury et al., 2009), UspA, UspC, and UspD were grouped in the same cluster (cluster green); while in a separate cluster (cluster blue) were grouped the UspE1; finally, the UspF and UspG were grouped into a third cluster (cluster red). Interestingly, phylogenetic analyses showed that the USPs from *Listeria* and *Lactobacillus* strains do not fit into any class described by Nachin et al. (2008) and formed, together the USPs of *Halomonas elongata, Mycobacterium tuberculosis*, and *Thermus thermophilus*, a distinct class that we have arbitrarily labeled UspL (cluster cyan).

This last class, in addition to the template and target sequences, includes also USPs from *Lb. plantarum* and *L. monocytogenes* strains, such as USP EHS84548 USP1 and USP Q8Y6V1_LSMO, involved in acid stress resistance.

**Figure 6:**
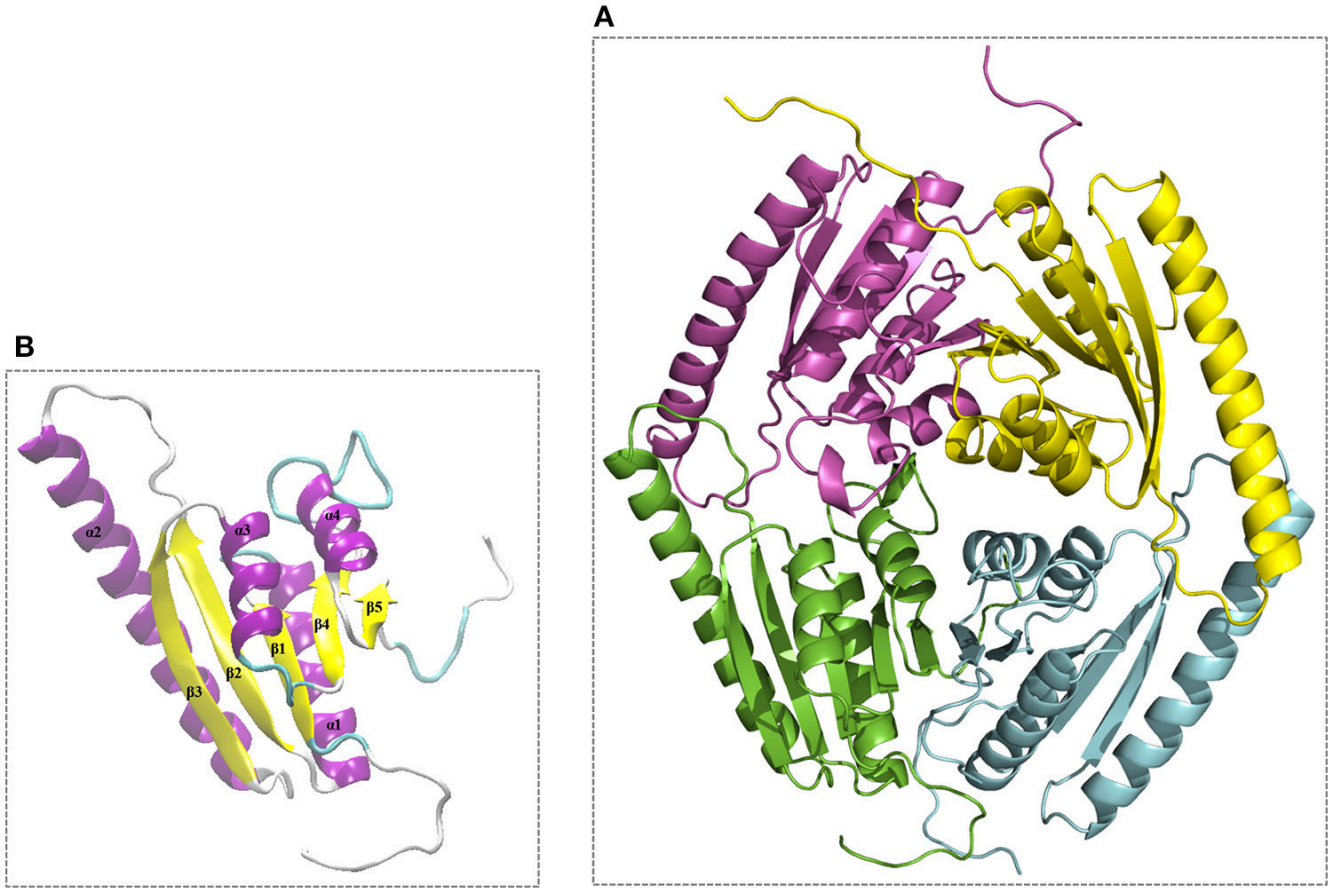
**Overall 3D structure of the Model_USP-691 from ***L. innocua*** ATCC 33090. (A)** 3D representation of tetrameric conformation containing the chain_A (green), chain_B (cyan), chain_C (magenta), and chain_D (yellow). **(B)**, representation of architecture having reference to the Rossmann-like α/β-fold with five parallel β-strands and four α-helices.

The modeled 3D structures of the USP belonging to *L. innocua* ATCC 33090 were stored as PDB output file and the best model (Model_USP-691) with a lower DOPE score (−0.247) was refined and used for both validation and interface analysis. Model_USP-691 (Figure 6A) was selected as the best to represent the quaternary structure of USPs with a homo-tetrameric conformation. Four monomers (chain A, B, C, and D) with an architecture similar to the Rossmann-like α/β-fold have five parallel β-strands and four α-helices (Figure 6B).

The Model_USP-691 showed a good accuracy in both the stereochemical properties and the absolute quality of the structure. The main chain-conformations for 95.9% aminoacid residues were allocated within the most favored region of Ramachandran plot, only two residues (GLN 169B and ASN 378C) were found in disallowed regions of the plot (Figure S2). The reliability of the selected Model_USP-691 was also confirmed by the good distribution of normalized QMEAN Z-score (−1.01) represented in Figure S3. Superposition of the Cα trace of the USP model (Model_USP-691) from *L. innocua* ATCC 33090 (magenta) and the template 3s3t (green) from *Lb. plantarum* was, as expected, very close, and small differences were observed in the N-terminal and C-terminal of helix α2.

### Interface analysis and structural alignments

**Figure 7:**
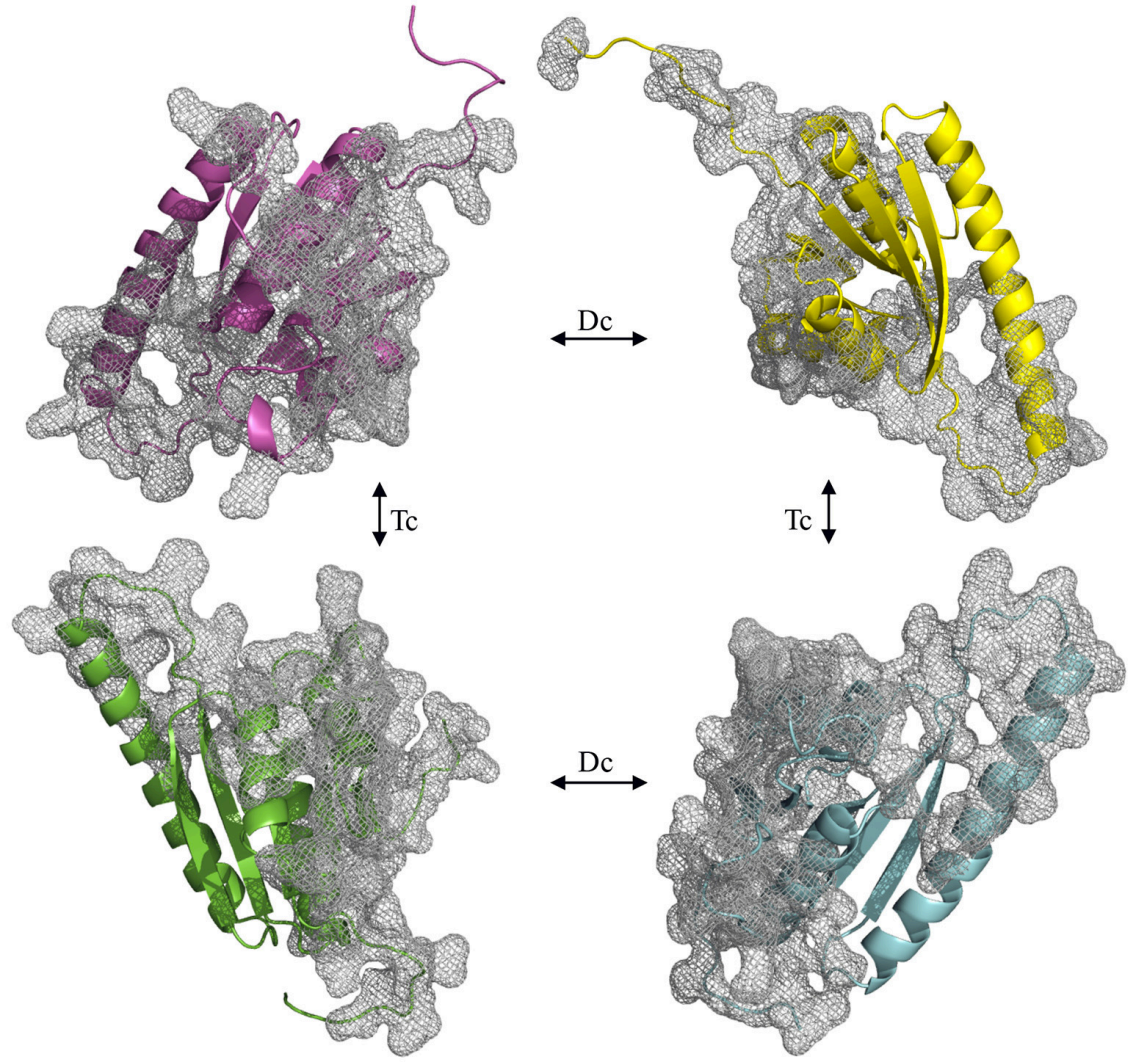
**Opened view of the Model_USP-691**. The grid represents the interfaces involved into dimeric (Dc) and tetrameric (Tc) contact. The chains are represented in green (chain_A), cyan (chain_B), magenta (chain_C), and yellow (chain_D).

To understand if the quaternary Model_USP-691 structure may have relevance in explaining some of the biological observations, the macromolecular interfaces of the predicted quaternary structures were explored in detail. We focused on the interfaces involved in both dimer and tetramer formation of model structure. Results obtained with PISA and POPSCOMP showed the presence of six interfaces (Table 3), but only four of them were thermodynamically favored, having a negative value in the solvation free energy gain (ΔG) from PISA. The interface area in the formation of dimers between chains A and B or C and D (Figure 7) was of about 1400 Å^2^ (PISA) or 1000 Å^2^ (POPSCOMP) involving a total of ~25% (PISA) or ~30% (POPSCOMP) of residues (Table S1) belonging to β5, α1, and α4 (Figure 6B). The tetrameric contacts between chains A and C or between B and D (Figure 7) covered an interface area of about 1000 Å^2^ (PISA) or 800 Å^2^ (POPSCOMP) including the 20% (PISA) or 22% (POPSCOMP) of residues (Table S1) dislocates in the regions α2, α3 and α4, and β2 (Figure 6B).

**Table 3:** **Interfaces analysis of Model_USP-691**.

**Interfaces**	**Structure 1**			**Structure 2**			**Iterface area (Å**^**2**^**)**		**ΔG (Kcal/mol)**	**N_HB**	**N_SB**
	**Chain**	**N_res**		**Chain**	**N_res**						
**PISA/PC**	**PISA/PC**	**PISA**	**PC**	**PISA/PC**	**PISA**	**PC**	**PISA**	**PC**	**PISA**		
1	A	39 (24%)	46 (29%)	B	43 (27%)	49 (31%)	1517	1116	−6.7	24	0
2	C	33 (21%)	36 (23%)	D	38 (24%)	41 (26%)	1259	924	−1.8	22	7
3	A	33 (21%)	36 (23%)	C	35 (22%)	39 (24%)	1164	839	−12.3	4	2
4	B	31 (19%)	33 (21%)	D	32 (20%)	38 (24%)	1099	830	−12.6	8	0
5	B	2 (1%)	2 (1%)	C	3 (2%)	3 (2%)	74	45	2.7	1	1
6	A	1 (0.6%)	1 (0.6%)	D	2 (1%)	2 (1%)	18	3	0.9	0	0

*PC, POPSCOMP server; N_res, number of residues; ΔG, solvation free energy gain; N_HB, number of hydrogen bonds; N_SB, number of salt bridges*.

**Figure 8:**
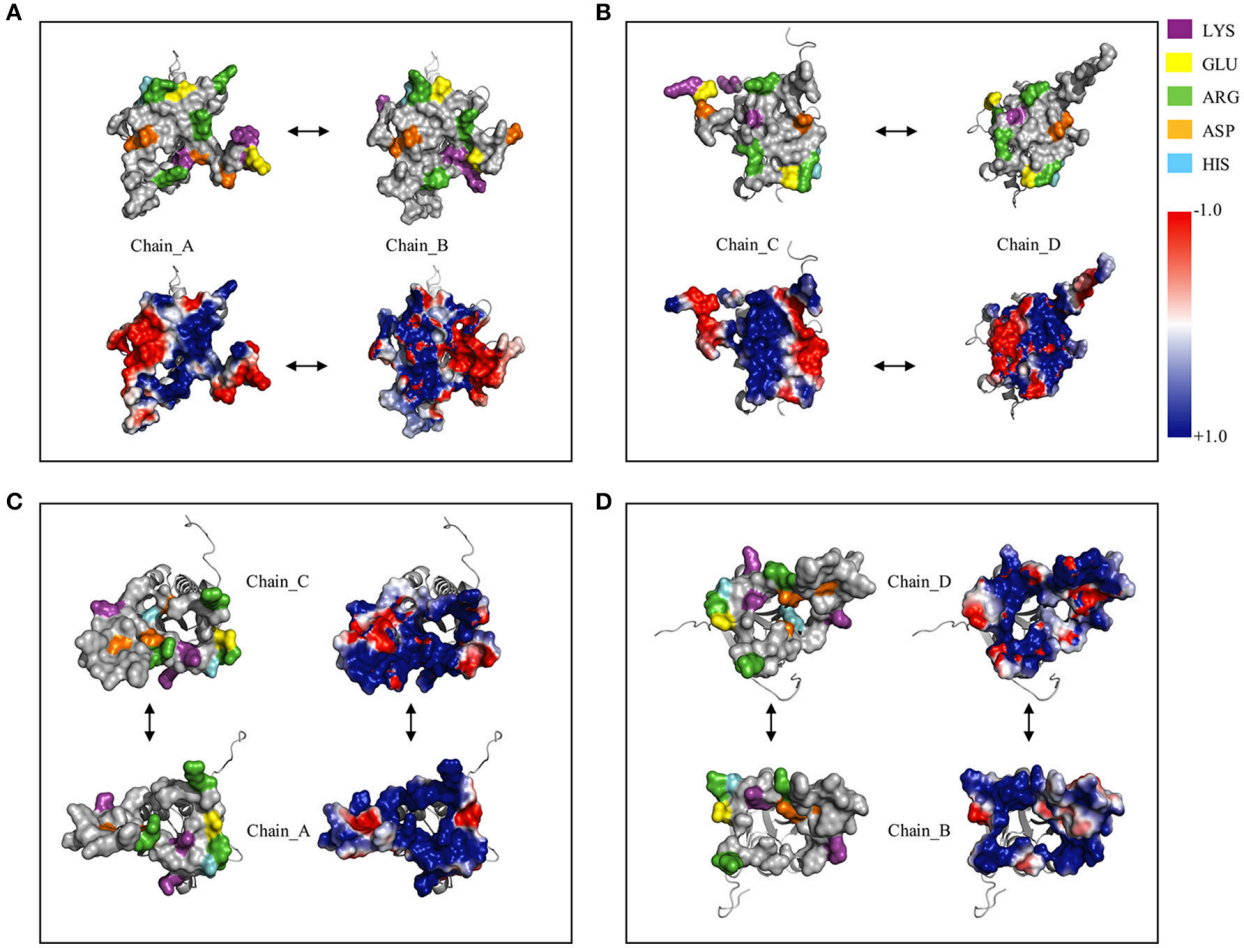
**Surface charged residues and electrostatic potential distribution at dimer and tetramer interfaces. (A)**, Complex between the chain_A and chain_B; **(B)**, complex between the chain_C and chain_D; **(C)**, complex between the chain_A and chain_C; **(D)**, complex between the chain_B and chain_D.

Figure 8 shows the surface charge density and electrostatic potential distribution at the interface between chains A and B (Figure 8A), C and D (Figure 8B), C and A (Figure 8C), and between chains D and B (Figure 8D). The complementary interfaces were covered by positive and negative charges as well as by hydrophobic patches. Moreover, the surface charges density highlighted that the dimer interfaces (A–B and C–D) were characterized by a hydrophobic core surrounded by a ring of polar residues. While, in the interfaces A–C and B–D a concave pocket positively charged can be noted.

**Figure 9:**
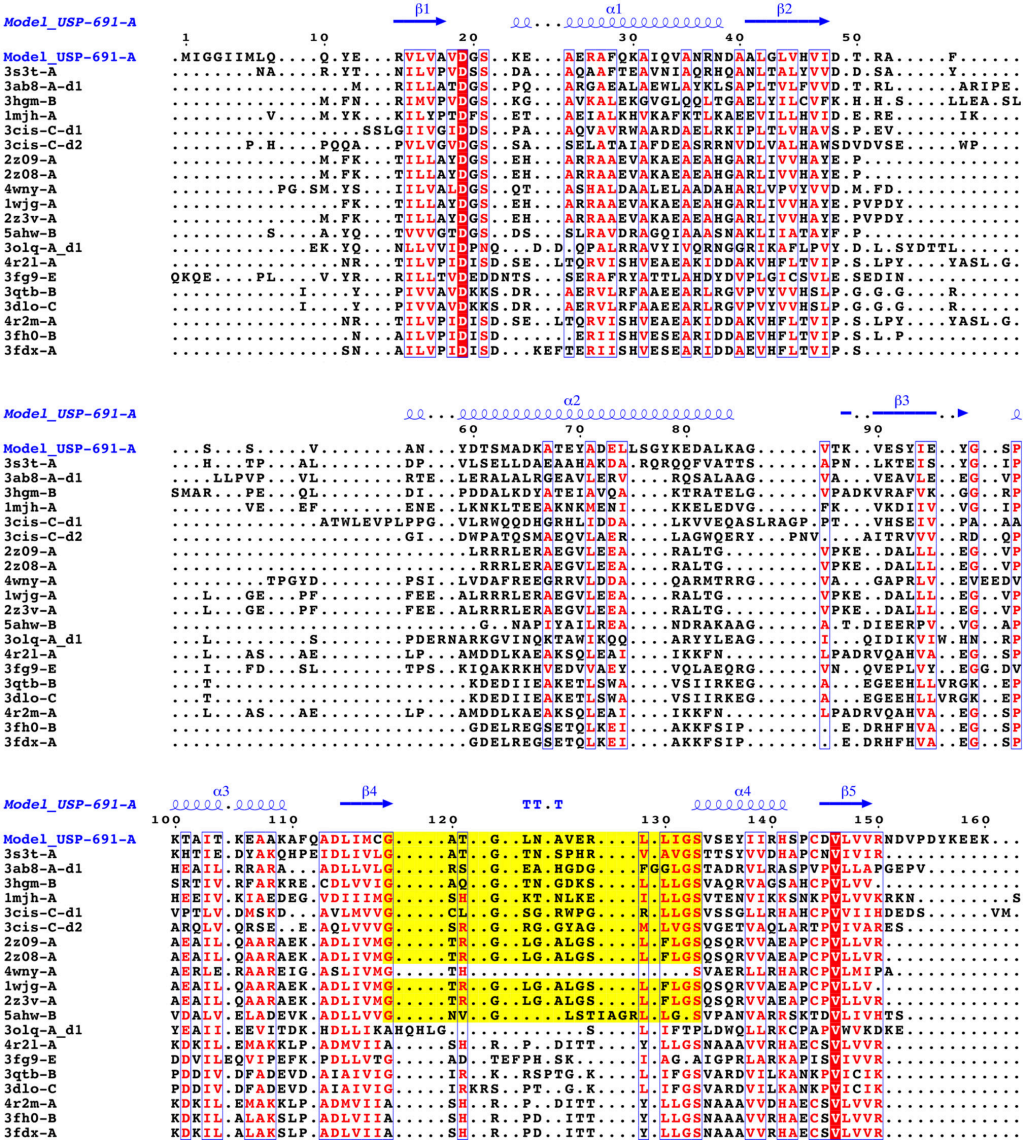
**Multiple structural alignment of sequences between the chain A of Model_USP-691 and proteins of homolog structures**. The number of domains are reported as d1or d2. The residues that are conserved in at least 60% of all sequences are highlighted in red. The conserved residues are represented in blue boxes. ATP-binding motif is in yellow.

The Model_USP-691-A (-A, chain A) was compared with the 3D structures of USPs available in the PDB by means of the DALI server (Table 4). Results showed that the Model_USP-691-A shared up to 34% of sequences identity overall 3D structure with the other USPs belonging both *Bacteria* and *Archaebacteria*. The overall folds were highly conserved in all USPs, some difference was detectable in the poorly conserved region α2 (Figure 8). The regions (α1, α4, and β5), involved into monomer-monomer interaction were highly conserved in all USPs. In addition, except for the fold α2, also the regions (α3, α4, and β5) of tetrameric contacts were conserved. Furthermore, the 31% (12/39) of residues involved in the formation of dimers Model_USP-691-A were conserved in at least 60% of the overall structures and 18% (6/33) of the residues involved into tetrameric association interfaces were conserved in 60% of the overall structures (Figure 9). Interestingly, the quaternary structure evidenced the occurrence of a loop containing the ATP-binding motif G-2X-G-9X-G-(S/T/N) characterized by three residues of glycine interspersed with two and nine amino acid residues between the first and the second glycine residues, respectively and with a serine/threonine/asparagine following the third glycine. The ATP-binding motif was also detectable into other 11 USP structures (Table 4, Figure 9).

**Table 4:** **3D-structures of USPs available in the PDB and searched with DALI server against the Model_USP-691-A**.

**PDB-chain-domain**	**Z-score**	**RMSD**	**lali^a^**	**nres^b^**	**% id**	**Protein (domain number)**	**Organism**	**Cofactor**	**ATP binding motif**		**References**
3s3t-A	26.2	0.4	143	145	31	Putative USP	*Lactobacillus plantarum*	ATP	Typical	G-2X-G-9X-GS	n/a
3ab8-A-d1	19.2	1.7	137	144	33	TTHA0350 (d1)	*Thermus thermophilus*	ATP	Similar	G-2X-G-10X-GS	Iino et al., 2011
3hgm-B	18.8	2.5	138	147	33	TEAD	*Halomonas elongata*	ATP	Typical	G-2X-G-9X-GS	Schweikhard et al., 2010
1mjh-A	18.7	2.0	139	143	31	MJ0577	*Methanococcus jannaschii*	ATP	Typical	G-2X-G-9X-GS	Zarembinski et al., 1998
3cis-C-d1	18.0	1.9	137	148	23	Rv2623 (d1)	*Mycobacterium tuberculosis*	ATP	Typical	G-2X-G-9X-GS	Drumm et al., 2009
3cis-C-d2	17.5	2.6	136	140	22	Rv2623 (d2)	*M. tuberculosis*	ATP	Typical	G-2X-G-9X-GS	Drumm et al., 2009
2z09-A	16.9	1.6	120	124	33	USP family	*T. thermophilus*	ACP	Typical	G-2X-G-9X-GS	n/a
2z08-A	16.8	1.7	119	123	34	USP family	*T. thermophilus*	ATP	Typical	G-2X-G-9X-GS	n/a
4wny-A	16.5	1.9	127	132	28	USP	*Burkholderia pseudomallei*	none	–		n/a
1wjg-A	16.4	1.8	124	135	30	Probable ATP binding	*T. thermophilus*	none	Typical	G-2X-G-9X-GS	n/a
2z3v-A	16.2	2.1	126	137	29	USP family	*T. thermophilus*	none	Typical	G-2X-G-9X-GS	n/a
5ahw-B	16.2	2.2	121	127	29	USP MSMEG_3811	*M. smegmatis*	cAMP/ATP	Typical	G-2X-G-9X-GS	Banerjee et al., 2015
3olq-A-d1	15.8	3.0	136	146	17	USP E (d1)	*Proteus mirabilis*	none	–		n/a
4r2l-A	15.7	2.8	135	142	25	YnaF	*Salmonella enterica*	ATP	–		Bangera et al., 2015
3fg9-E	15.5	2.5	135	150	26	UspA family	*Lb. plantarum*	none	–		n/a
3qtb-B	15.5	2.5	126	133	26	AF0826	*Archaeoglobus fulgidus*	dAMP	–		Tkaczuk et al., 2013
3dlo-C	15.4	2.2	124	133	26	AF0826	*A. fulgidus*	none	–		Tkaczuk et al., 2013
4r2m-A	15.3	2.8	133	141	25	YnaF	*S. enterica*	ANP	–		Bangera et al., 2015
3fh0-B	15.3	2.7	124	125	27	Putative USP KPN_01444	*Kleibsiella pneumonia*	ADP	–		n/a
3fdx-A	15.0	2.6	123	127	27	USP F	*K. pneumonia*	ATP	–		n/a
–	–										
3ab8-A-d2	13.8	2.1	111	115	24	TTHA0350 (d2)	*T. thermophilus*	ATP	Degenerated	G-8X-GS	Iino et al., 2011
3olq-A-d2	12.8	3.4	131	159	14	USP E (d2)	*P. mirabilis*	none	–		n/a

a *Matching residues*.

b *Total number of residues*.

## Discussion

In this study, *L. innocua* ATCC 33090 was used as a surrogate of the pathogen species *L. monocytogenes*, to investigate the effect of sub-lethal acid pH on the growth and protein expression. In agreement with results obtained for the pathogenic microorganism (Begley and Hill, 2015), *L. innocua* was able to quickly adapt to metabolic pathways in response to acid stress, modifying the expression of a protein subset.

In fact, the results highlighted that under acid stress conditions, 19 gene products were at least two-fold up-regulated in *L. innocua* during the growth (exponential phase).

Some of the upregulated proteins identified in the current study have been already associated to acid stress response as well as to other stress factors. The SSDH is the second enzyme of the γ-aminobutyrate (GABA) shunt pathway (Zhu et al., 2010). In the GABA shunt pathway, GABA is firstly converted to succinate-semialdehyde (SSA) by means of GABA aminotransferase (GABA-AT) and then oxidized by SSDH to succinate with formation of CO_2_. The GABA shunt can operate as an alternative pathway to provide succinate in some steps of the tricarboxylic acid (TCA) cycle in bacteria, including *L. monocytogenes*, that lacks of a complete set of genes necessary for the TCA cycle (Glaser et al., 2001). Moreover, GABA shunt may be an important source of nitrogen in certain bacteria (Schneider et al., 2002), as well as may play a role in acid tolerance in *L. monocytogenes* (Abram et al., 2008).

The CTC protein belongs to the L25 ribosomal protein family and is involved in the adaptation of *L. monocytogenes* to osmotic stress in the absence of osmoprotectants (Duché et al., 2002; Gardan et al., 2003). Moreover, the results in our study also highlighted the up-regulation of a specific USP during the growth (exponential phase) in acid conditions. It is likely to suspect that this specific protein -together with other acid stress proteins—was involved in the response to acid stress during the growth phase of *L. innocua*. A link between the USP up-regulation and the acid stress condition was clearly revealed. In fact, the specific USP was up-regulated or down-regulated as response to acid stress condition or to the restoration of conventional conditions, respectively.

To the best of our knowledge, only Seifart Gomes et al. (2011) highlighted the importance of USP in response to the acid stress in pathogenic *L. monocytogenes*. These authors revealed a clear role of USP in the survival of cells showing that the resistance of *usp*-deleted mutants was significantly reduced compared to the wild stains. The role of USPs in response to several stress conditions was better elucidated in other bacteria, including *Escherichia coli* (Gustavsson et al., 2002; Nachin et al., 2005) and *Salmonella typhimurium* (Liu et al., 2007; Bangera et al., 2015), *Haemophilus influenzae* (Fleischmann et al., 1995; Sousa and McKay, 2001), *Mycobacterium tuberculosis* (O'Toole and Williams, 2003; Drumm et al., 2009), *Pseudomonas aeruginosa* (Boes et al., 2008), and *Lactobacillus plantarum* (Licandro-Seraut et al., 2008; Gury et al., 2009). In all cases, the expression of USP has been associated to the arrest of cellular growth in response to prolonged stress (Hingley-Wilson et al., 2010). Nyström and Neidhardt (1992, 1994) showed that the survival of *E. coli* was reduced in the *uspA*-mutated strains. Likewise, the mutation of *uspA* gene reduced the survival of *S. thyphimurium* to carbon or phosphorous (Liu et al., 2007). Moreover, the USP PA3309 and PA4352 were essential for survival of *Pseudomonas aeruginosa* under anaerobic conditions (Boes et al., 2006; Schreiber et al., 2006). *Usp*-deleted mutants of *Burkholderia glumae* showed a significant reduction of survival when compared to wild-type strains after the heat-shock stress (Kim et al., 2012).

Herein, USP over-expression was associated with the cellular growth arrest of *L. innocua*, both in the presence and in the absence of acid. However, when the strain was cultivated in acid conditions, unexpectedly USP was over-expressed during the exponential phase. This finding suggests that USP in *Listeria* could play a crucial role in the response to acid stress during the exponential growth, and represents an important advance in the knowledge of the functional role of the USP family. For this purpose, the phylogenetic analysis offered an interesting information, showing that USPs of *Listeria* were distant from other previously characterized USPs (Nachin et al., 2008) belonging to *E. coli, Salmonella*, and *H. influeanzae*. In detail, all the USPs from *Lactobacillus* spp. and *Listeria* spp. (including template and target) clustered in a separate and heterogeneous class, arbitrarily called USPL. Therefore, we can assume that the USP from the USPL class could play a different role in the stress response than USPs grouped in other classes.

Structural and biochemical studies suggest a wide array of functions of USPs. Anyway, few USP structures are available in PDB and there is little structural information is available for most of them. We found that of the 20 proteins with a sequence similar (Z-score significance ≥15.0) to the Model_USP-691-A, only for about 50% of them there is available structural information (Table 4). Generally, USPs have a structure typical of a Rossmann-like α/β-fold having five-stranded parallel β-sheet surrounded by four α-helices that homo-dimerize, in an antiparallel fashion via the fifth β-strand on each subunit (Zarembinski et al., 1998; Schweikhard et al., 2010; Tkaczuk et al., 2013). Moreover, proteins such as TTHA0350, Rv2623, and YdaA were found to display two USP domains (Drumm et al., 2009; Iino et al., 2011; Bangera et al., 2015). Both single and double USP domain-containing proteins may assembly to form either tetrameric structure characterized by four USP domains or a homo-dimeric of two chains with two USP domains. In this work, the homology modeling technique used supports a homo-tetrameric structure of USP (Figure 6B). Bioinformatics analyses were addressed to the comparison of the modeled structures and of the interface regions with previously characterized USP members. The Model_USP-691-A shared a sequences identity of 17–34% with a Z-score ranging from 15.0 to 26.2 (Table 4) when compared to resolved USP structures deposited in PDB. Moreover, the model reliability was confirmed by the presence of highly conserved folding regions (Figure 9), especially those represented by the β-strands. The β-strands 1–5 contained residues with hydrophobic properties playing an important role in the protein folding. These regions are involved in the formation of a stable β-sheet, described by Iino et al. (2011) as a typical USP molecular core. Interestingly, the hydrophobic properties of high structurally and sequentially conserved residues (V^146^-L^147^-V^148^-V^149^) of the β-strand 5 seem to have an essential role into monomer-monomer interface formation (Figures 6B, 7, 9). In particular the residue V^146^, which is conserved in all the USP structures and sequences observed so far (Figure 9 and Figure S1), could play a key role into monomer-monomer contacts. Schweikhard et al. (2010) found that the residues V^144^ and V^146^ of TeaD protein, corresponding to V^146^ and V^148^ of our Model_USP-691-A, were involved into monomer-monomer contacts through hydrogen bonds. We also predicted the presence of hydrogen bonds between the residues V^146^ and V^148^ of chain A with the residues V^148^ and V^146^ of chain B respectively. The region α4 took part in the formation of dimers with the involvement of 4 residues (E^136^-2X-I^139^-R^140^-H^141^; where X indicate the number of residues between) as well, but only the residues I^139^ and H^141^ resulted conserved in the sequence. Probably these residues, together with the others found conserved in the structure and sequences of USPs, may function as hot spots in monomer–monomer interface assembly (Figure 9). On the base of the herewith-collected structural and sequence information, it would seem that the dimeric arrangement of Model_USP-691 is a plausibly correct assembly. This hypothesis is also supported by the analysis of the composition of interfaces. We found the presence of a large interface area (~1400 Å^2^) between the chains A and B or C and D of model, compatible in size with the interfaces (740–1900 Å^2^) of other crystallographic resolved USPs. In addition, a complementary electrostatic charge distribution was found at the interface. Moreover, the negative value of solvation free energy gain upon formation of the interface, as well as the high number of hydrogen bounds found at the interface, suggest a favorable interaction between the USP monomers. The proposed USP assembly (Figure 7) suggests that the dimer could have a crucial functional role. Although the biological function of these dimers is still unknown, this observation is supported by the recovery of proteins (TTHA0350, Rv2623, YdaA) with two tandem USP domains. In the work conducted by Schweikhard et al. (2010) the USP protein TeaD showed a dimeric state as assessed by SEC (Size exclusion chromatography) and Blue Native PAGE analyses. The same authors, showed that when ATP was added to TeaD, a tetrameric state was also observable, concluding that ATP significantly contributed to the stabilization of the molecular tetrameric conformation. Other authors highlighted the putative role of ATP in the contact between tetrameric assembly of USPs (Sousa and McKay, 2001). Recently, Bangera et al. (2015) showed that both single domain USP YnaF and two domains USP YdaA from *S. typhimurium* had a tetrameric or dimeric organization, respectively, and that together they could bind ATP. In fact, ATP or nucleotide binding USPs display a conserved ATP-binding motif G-2X-G-9X-G(S/T-N). We found this motif present in the structure of Model_USP-691-A, and structurally conserved compared to the more closely related USPs (Figure 9). Notably, the presence of a positively charged pocket indicated an electrostatic compatibility with the ATP molecule. The residues H^141^, S^133^, L^128^, P^99^, A^67^, I^48^ were structurally and sequentially conserved, pointing a role into tetrameric conformation of model. Overall, this information supports the tetrameric assembly shown by Model_USP-691. The possible ATP-binding property would be fundamental for the USP assembly and consequently for the protein function. According to a previous biochemical study, USPs from other bacterial species have been shown to display ATPase activity (Zarembinski et al., 1998). More recently, Bangera et al. (2015) reported the USP YdaA (PDB ID: 4r2j) of *S. typhimurium* showing ATPase activity and contain an ATP binding motif; in contrast, an additional USP (YnaF, PDB ID: 4r2l) protein did not show any ATPase activity, but was able to bind ATP though lacking the specific ATP binding motif. Therefore, the biochemical and biological function of the USPs could rely on an ATP dependent-factor, which is likely to be linked to a specific energetic state of the cell.

In conclusion, based on the current structural prediction, *Listeria* USP might be deemed as a new type of ATP-binding USP. Contrarily to USP-types involved into growth arrests, the USP of *L. innocua* could have a key role in the response to acid stress during the exponential growth phase.

## Author contributions

PT: design of the work, analysis and interpretation of the microbial and proteomic data, drafting the work; MS: interpretation of the data, drafting the work, and revising it critically; RC: conception of the work, drafting the work, and revising it critically; ES: involved in experimental designing; LT: analysis of the microbial growth; GPi: analysis and interpretation of mass spectrometry data; GPa: design of the work, execution of 2-DE and bioinformatic analyses, interpretation of data, drafting and revising the work. FF: design of the bioinformatics approaches, agreement to be accountable for all aspects of the work in ensuring that questions related to the accuracy or integrity of any part of the work are appropriately investigated and resolved.

### Conflict of interest statement

The authors declare that the research was conducted in the absence of any commercial or financial relationships that could be construed as a potential conflict of interest.
