# Supplementary material for: Homology-Based Modeling of Universal Stress Protein from Listeria innocua Up-Regulated under Acid Stress Conditions
Source: Front Microbiol. 2016 Dec 20;7:1998. doi: 10.3389/fmicb.2016.01998 (PMC5168468; doi:10.3389/fmicb.2016.01998)
Supplement: Supplementary file 1 [file Image1.PDF]

Supplementary Material

Homology-based modeling of Universal Stress Protein from *Listeria innocua* up-regulated under acid stress conditions

Patrizio Tremonte, Mariantonietta Succi, Raffaele Coppola, Elena Sorrentino, Luca Tipaldi, Gianluca Picariello, Gianfranco Pannella*, Franca Fraternali

*** Correspondence:** Gianfranco Pannella: gianfranco.pannella@unimol. Supplementary Figures

Supplementary Figure 1

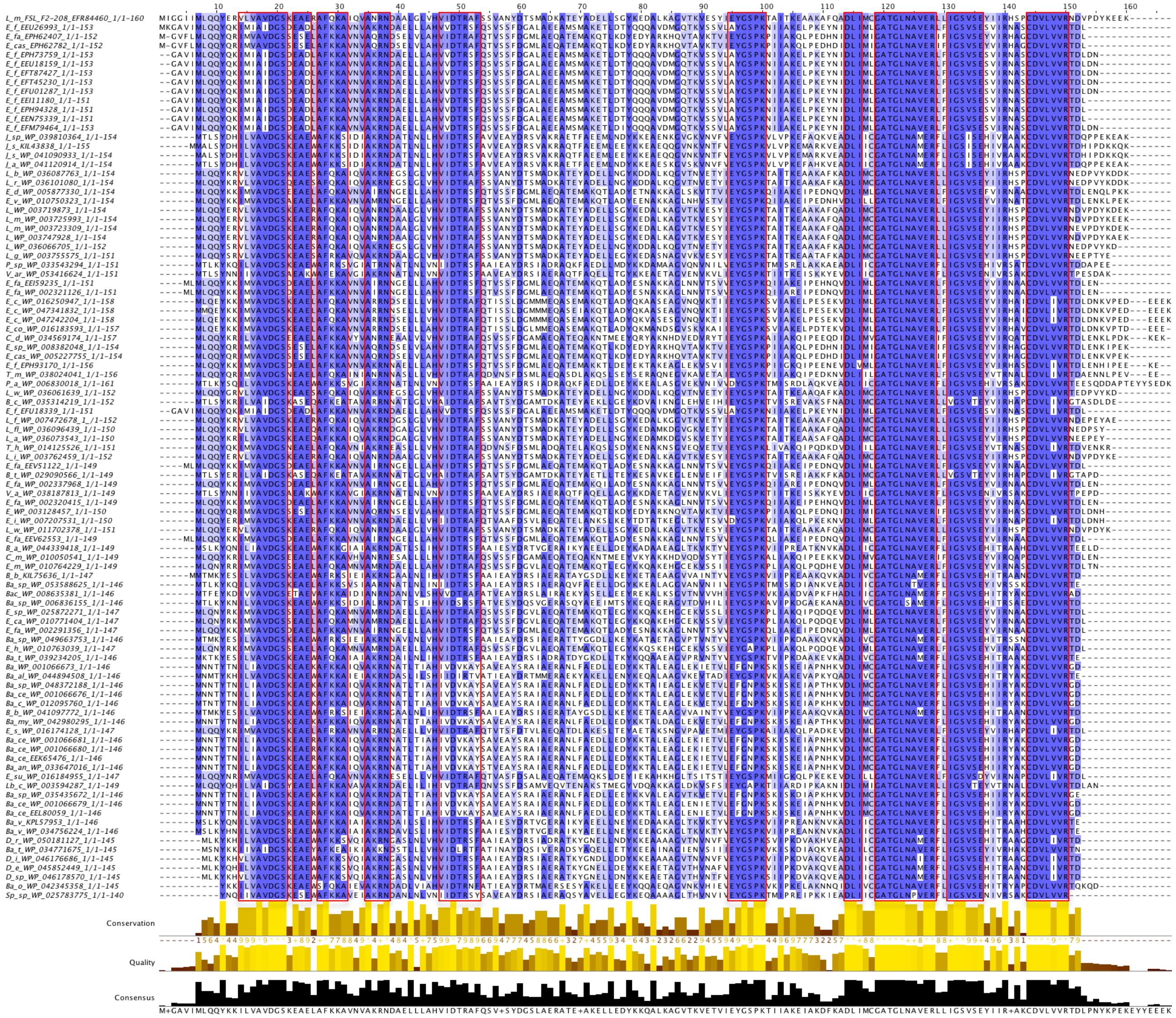

Figure S1. Multi-sequence alignment between the target sequence and homologous sequences retrieved from a non-redundant database using BLASTp.
