# Supplementary material for: Homology-Based Modeling of Universal Stress Protein from Listeria innocua Up-Regulated under Acid Stress Conditions
Source: Front Microbiol. 2016 Dec 20;7:1998. doi: 10.3389/fmicb.2016.01998 (PMC5168468; doi:10.3389/fmicb.2016.01998)
Supplement: Supplementary file 3 [file Image3.PDF]

Supplementary Material

Homology-based modeling of Universal Stress Protein from *Listeria innocua* up-regulated under acid stress conditions

Patrizio Tremonte, Mariantonietta Succi, Raffaele Coppola, Elena Sorrentino, Luca Tipaldi, Gianluca Picariello, Gianfranco Pannella*, Franca Fraternali

*** Correspondence:** Gianfranco Pannella: gianfranco.pannella@unimol.it

Supplementary Figure 3

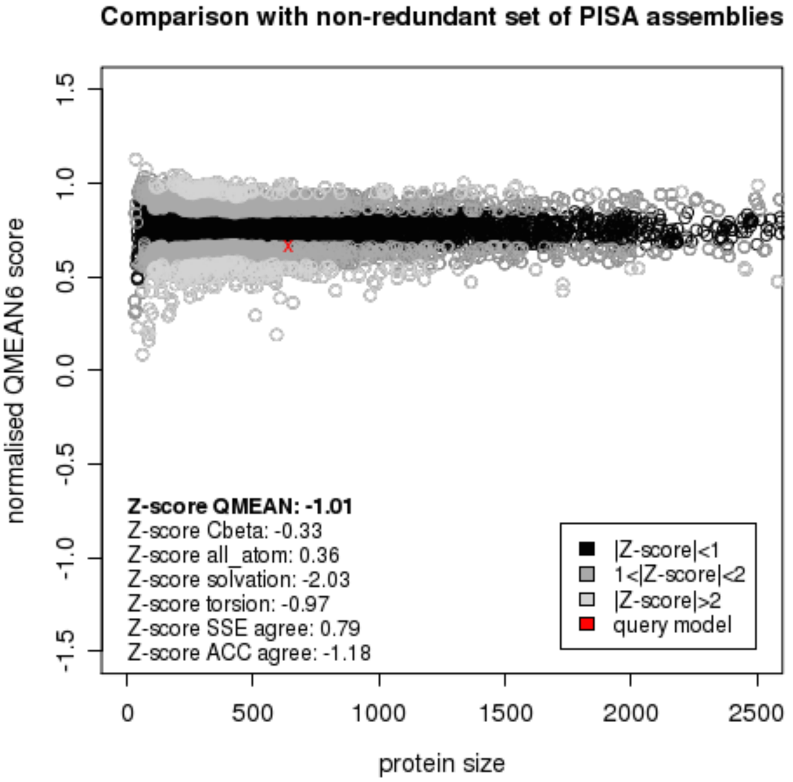

Figure S3. Q Mean z-score of the Model_USP-691.
