# Supplementary material for: Homology-Based Modeling of Universal Stress Protein from Listeria innocua Up-Regulated under Acid Stress Conditions
Source: Front Microbiol. 2016 Dec 20;7:1998. doi: 10.3389/fmicb.2016.01998 (PMC5168468; doi:10.3389/fmicb.2016.01998)
Supplement: Supplementary file 4 [file Table1.PDF]

Supplementary Material

Homology-based modeling of Universal Stress Protein from *Listeria innocua* up-regulated under acid stress conditions

Patrizio Tremonte, Mariantonietta Succi, Raffaele Coppola, Elena Sorrentino, Luca Tipaldi, Gianluca Picariello, Gianfranco Pannella*, Franca Fraternali

*** Correspondence:** Gianfranco Pannella: gianfranco.pannella@unimol.it

1 Supplementary Tables

Table S1 List of residues at interfaces.

Complex A-B																							
Structure1												Structure2											
			POPSCOMP				PISA								POPSCOMP				PISA				
##	Res	Chain	ResNr	Phob	Phil	Tot	ResNr	HSDC	ASA	BSA	Δ G	##	Res	Chain	ResNr	Phob	Phil	Tot	ResNr	HSDC	ASA	BSA	Δ G
4	GLY	A	4	0.00	0.44	0.44						1	MET	B	161	3.13	11.58	14.71	161		196.36	29.38	-0.22
5	ILE	A	5	38.11	0.00	38.11	5		130.64	59.19	0.95	2	ILE	B	162	1.00	0.00	1.00	162		83.13	4.02	0.06
6	ILE	A	6	13.43	1.89	15.31	6		171.59	22.89	0.20	3	GLY	B	163	6.48	20.59	27.07	163		77.46	29.12	-0.31
7	MET	A	7	73.74	0.26	74.01	7		133.28	90.24	1.91	4	GLY	B	164	8.06	2.68	10.75	164		82.39	9.05	0.14
8	LEU	A	8	71.66	14.56	86.22	8		188.80	85.33	0.78	5	ILE	B	165	0.00	0.16	0.15					
9	GLN	A	9	43.75	73.74	117.49	9	H	140.76	114.68	-0.82	7	MET	B	167	28.52	0.00	28.53	167		87.41	46.22	1.41
10	GLN	A	10	1.60	1.46	3.06	10		96.83	1.60	0.03	8	LEU	B	168	4.23	1.52	5.74	168		171.89	15.55	0.19
11	TYR	A	11	3.42	3.89	7.31	11		49.34	25.49	0.22	9	GLN	B	169	31.21	65.45	96.66	169	H	158.64	100.56	-0.73
29	GLN	A	29	0.23	0.45	0.68	29		86.05	5.26	0.06	10	GLN	B	170	3.06	1.00	4.06	170		62.00	2.79	0.04
30	LYS	A	30	19.60	18.62	38.22	30		53.35	43.02	-0.25	11	TYR	B	171	13.89	9.78	23.68	171		64.77	46.24	0.34
32	ILE	A	32	0.16	0.00	0.15						26	ARG	B	186	0.07	0.00	0.07					
33	GLN	A	33	8.53	27.31	35.84	33		82.33	48.10	-0.20	29	GLN	B	189	0.50	0.21	0.72	189		73.19	9.76	0.05
36	ASN	A	36	0.00	0.39	0.39	36		56.60	4.95	-0.06	30	LYS	B	190	11.57	1.39	12.97	190		40.21	25.99	0.42
37	ARG	A	37	20.18	66.83	87.01	37	H	159.09	123.10	-0.93	32	ILE	B	192	1.42	0.07	1.49	192		16.18	10.39	0.17
38	ASN	A	38	0.41	4.05	4.47	38		23.11	17.12	-0.25	33	GLN	B	193	19.31	53.51	72.82	193	H	82.55	77.43	-0.35
111	PHE	A	111	46.98	0.00	46.98	111		161.61	56.37	0.90	34	VAL	B	194	1.01	0.08	1.09	194		27.79	5.37	0.09
114	ASP	A	114	0.01	0.61	0.62	114		47.06	4.54	-0.08	36	ASN	B	196	1.88	6.51	8.38	196		39.52	23.28	0.00
120	ALA	A	120	0.05	0.00	0.05						37	ARG	B	197	35.89	37.86	73.77	197	H	147.01	111.48	-0.33
128	ARG	A	128	0.00	0.48	0.48	128		214.40	0.86	-0.01	38	ASN	B	198	0.60	7.02	7.63	198	H	17.05	12.81	-0.19
129	LEU	A	129	11.76	0.00	11.76	129		90.54	26.61	0.43	85	ALA	B	245	0.43	0.00	0.42	245		26.55	2.35	0.04
130	LEU	A	130	56.77	0.48	57.26	130		142.18	43.71	0.61	108	ALA	B	268	4.21	3.65	7.85	268	H	63.30	26.00	-0.05
131	ILE	A	131	19.95	0.41	20.36	131		50.43	43.57	0.70	109	LYS	B	269	0.40	0.36	0.76	269		144.81	0.33	0.01
135	SER	A	135	0.02	0.22	0.23						111	PHE	B	271	39.05	1.85	40.90	271		178.10	61.43	0.83
136	GLU	A	136	0.10	1.01	1.12	136		54.09	2.96	-0.03	112	GLN	B	272	0.05	0.04	0.09	272		56.18	0.17	0.00
138	ILE	A	138	0.01	0.19	0.20						114	ASP	B	274	0.07	2.59	2.66	274		55.03	12.39	-0.21
139	ILE	A	139	31.48	5.16	36.65	139	H	73.80	71.46	0.77	115	LEU	B	275	0.12	0.00	0.12					
140	ARG	A	140	16.16	22.29	38.45	140	H	158.54	88.47	-0.82	130	LEU	B	290	49.20	0.17	49.37	290		129.38	39.30	0.62
141	HIS	A	141	4.09	2.71	6.80	141		86.38	14.36	-0.14	131	ILE	B	291	30.32	0.33	30.66	291		71.66	53.94	0.86
142	SER	A	142	3.38	8.30	11.68	142	H	18.44	17.88	-0.17	135	SER	B	295	0.00	0.12	0.11					
143	PRO	A	143	10.87	17.10	27.96	143	H	85.05	40.95	-0.19	136	GLU	B	296	2.87	6.45	9.33	296		102.26	17.90	0.01
144	CYS	A	144	1.95	3.26	5.20	144		24.95	12.89	0.09	138	ILE	B	298	0.00	0.17	0.17					
145	ASP	A	145	14.92	41.51	56.43	145	H	80.51	68.97	-0.54	139	ILE	B	299	32.83	5.60	38.43	299	H	70.63	68.79	0.69
146	VAL	A	146	4.88	12.46	17.35	146	H	35.24	35.12	-0.23	140	ARG	B	300	19.10	31.23	50.32	300	H	181.13	96.16	-0.73
147	LEU	A	147	22.46	0.43	22.90	147		64.59	41.98	0.67	141	HIS	B	301	2.33	2.37	4.70	301		69.94	12.64	0.15
148	VAL	A	148	34.71	14.49	49.20	148	H	60.10	58.03	0.34	142	SER	B	302	3.94	10.15	14.09	302	H	21.50	18.88	-0.20
149	VAL	A	149	7.09	0.75	7.85	149		12.39	9.88	0.16	143	PRO	B	303	7.17	12.92	20.10	303		92.17	18.86	-0.15

Continued

Complex A-B *Continued*

Structure1												Structure2											
			POPSCOMP				PISA								POPSCOMP				PISA				
##	Res	Chain	ResNr	Phob	Phil	Tot	ResNr	HSDC	ASA	BSA	Δ G	##	Res	Chain	ResNr	Phob	Phil	Tot	ResNr	HSDC	ASA	BSA	Δ G
150	ARG	A	150	42.32	56.95	99.26	150	H	129.31	98.23	0.02	144	CYS	B	304	2.58	4.72	7.31	304		30.23	15.17	0.07
151	ASN	A	151	1.93	10.74	12.66	151	H	71.34	19.88	-0.28	145	ASP	B	305	15.73	46.08	61.82	305	H	82.49	76.74	-0.49
152	ASP	A	152	6.46	8.17	14.63	152		57.73	22.70	0.12	146	VAL	B	306	4.88	13.83	18.71	306	H	39.74	39.47	-0.21
153	VAL	A	153	0.16	1.29	1.45	153		78.63	10.43	-0.12	147	LEU	B	307	17.60	0.49	18.08	307		61.23	39.72	0.64
154	PRO	A	154	3.45	0.46	3.90	154		136.73	14.50	0.20	148	VAL	B	308	35.01	12.52	47.53	308	H	65.02	63.00	0.38
155	ASP	A	155	0.07	0.00	0.07						149	VAL	B	309	8.12	0.74	8.86	309		23.40	14.36	0.23
156	TYR	A	156	35.33	5.86	41.19	156		151.14	52.92	0.54	150	ARG	B	310	27.47	64.24	91.69	310	H	115.21	94.12	-1.30
157	LYS	A	157	0.17	0.74	0.90	157		151.98	0.12	0.00	151	ASN	B	311	12.47	14.58	27.04	311	H	74.50	38.80	-0.01
158	GLU	A	158	0.26	0.04	0.31						155	ASP	B	315	0.00	0.03	0.02					
160	LYS	A	160	14.35	25.91	40.27	160	H	215.79	39.62	-1.11	156	TYR	B	316	21.97	1.27	23.24	316		142.03	31.98	0.51
												157	LYS	B	317	9.50	1.52	11.01	317		161.43	19.77	0.20
												159	GLU	B	319	12.36	17.29	29.65	319	H	71.93	22.13	-0.19
												160	LYS	B	320	73.39	10.19	83.59	320	H	205.05	52.62	0.75

Complex C-D

Structure1

			POPSCOMP				PISA				
##	Res	Chain	ResNr	Phob	Phil	Tot	ResNr	HSDC	ASA	BSA	Δ G
8	LEU	C	328	38.04	0.00	38.04	328		136.38	45.26	0.72
9	GLN	C	329	25.96	64.57	90.53	329	H	177.86	96.22	-0.78
10	GLN	C	330	1.67	10.51	12.18	330		93.15	26.60	-0.36
11	TYR	C	331	9.29	12.56	21.84	331		74.81	45.19	0.11
29	GLN	C	349	0.06	2.33	2.39	349		92.52	9.91	-0.11
30	LYS	C	350	21.18	20.67	41.85	350	HS	52.62	31.88	-0.36
33	GLN	C	353	7.20	32.24	39.44	353	H	69.78	48.26	-0.34
34	VAL	C	354	0.92	0.00	0.92	354		27.29	4.34	0.07
37	ARG	C	357	14.17	85.81	99.99	357	H	186.82	117.62	-0.97
38	ASN	C	358	0.21	4.36	4.57	358		32.79	14.26	-0.17
84	LYS	C	404	3.57	8.18	11.76	404		162.85	22.03	-0.51
115	LEU	C	435	1.31	0.00	1.31	435		7.87	6.03	0.10
117	MET	C	437	0.10	0.00	0.10					
130	LEU	C	450	50.72	0.20	50.90	450		134.43	39.36	0.63
131	ILE	C	451	25.90	0.27	26.17	451		56.90	43.94	0.70
135	SER	C	455	0.01	0.05	0.06					
136	GLU	C	456	11.05	29.44	40.51	456	HS	64.52	38.18	-0.08
139	ILE	C	459	30.12	3.11	33.24	459		77.93	77.35	0.96
140	ARG	C	460	15.26	36.12	51.38	460	HS	176.44	81.55	-0.20
141	HIS	C	461	3.06	1.87	4.94	461		91.70	16.80	0.22
142	SER	C	462	1.60	7.67	9.28	462	H	21.33	19.14	-0.22
143	PRO	C	463	7.25	12.35	19.60	463		93.12	22.53	-0.08
144	CYS	C	464	1.09	2.46	3.55	464		27.51	9.76	0.07
145	ASP	C	465	17.11	50.69	67.79	465	HS	88.71	80.23	-0.58
146	VAL	C	466	3.96	15.33	19.30	466	H	39.80	39.80	-0.31
147	LEU	C	467	43.88	0.63	44.50	467		68.95	64.93	1.04
148	VAL	C	468	10.09	10.14	20.23	468	H	39.93	38.25	0.07
149	VAL	C	469	4.74	0.60	5.34	469		4.67	4.33	0.07
150	ARG	C	470	43.64	25.61	69.24	470	H	125.59	74.22	0.32
151	ASN	C	471	23.43	68.48	91.92	471	H	107.39	71.33	-0.78
153	VAL	C	473	1.52	0.00	1.51	473		75.58	1.82	0.03
154	PRO	C	474	24.66	0.10	24.75	474		132.81	32.13	0.51
155	ASP	C	475	0.00	0.07	0.07					
156	TYR	C	476	0.00	2.36	2.35	476		211.90	0.98	-0.01
159	GLU	C	479	14.65	51.24	65.88	479		145.15	68.44	-0.67
160	LYS	C	480	3.02	8.13	11.15	480		239.29	22.32	-0.09

Structure2

			POPSCOMP				PISA				
##	Res	Chain	ResNr	Phob	Phil	Tot	ResNr	HSDC	ASA	BSA	Δ G
1	MET	D	481	12.77	0.00	12.78	481		235.94	22.58	0.36
5	ILE	D	485	16.18	5.49	21.67	485		149.16	31.90	0.14
6	ILE	D	486	16.14	0.62	16.76	486		158.86	7.85	0.13
7	MET	D	487	12.40	3.27	15.67	487		137.71	17.30	0.27
8	LEU	D	488	28.91	1.06	29.97	488		146.43	21.08	0.34
9	GLN	D	489	35.21	34.66	69.86	489	H	163.41	100.42	-0.17
10	GLN	D	490	4.29	2.71	7.00	490		71.10	5.88	0.05
11	TYR	D	491	14.78	11.46	26.25	491	H	66.22	49.26	0.27
26	ARG	D	506	0.34	9.38	9.72	506	H	49.26	12.81	-0.07
29	GLN	D	509	0.09	0.02	0.11	509		23.54	0.17	0.00
30	LYS	D	510	22.73	10.80	33.53	510	HS	55.56	40.33	-0.08
32	ILE	D	512	0.64	0.00	0.63	512		27.55	3.02	0.05
33	GLN	D	513	7.32	30.12	37.43	513	H	73.04	57.76	-0.20
34	VAL	D	514	0.67	0.00	0.67	514		14.06	2.18	0.03
36	ASN	D	516	0.02	0.33	0.35	516		59.80	4.23	-0.07
37	ARG	D	517	17.05	17.41	34.45	517	H	162.83	91.30	-0.43
38	ASN	D	518	0.73	6.35	7.08	518		27.94	17.89	-0.17
111	PHE	D	591	1.02	0.00	1.02	591		169.45	0.94	0.02
113	ALA	D	593	0.01	0.20	0.21					
114	ASP	D	594	2.09	2.73	4.82	594	H	44.61	14.78	0.08
115	LEU	D	595	1.47	0.03	1.50	595		7.37	4.19	0.07
116	ILE	D	596	0.00	0.02	0.02					
117	MET	D	597	0.43	0.00	0.42					
130	LEU	D	610	46.07	0.37	46.44	610		129.44	38.47	0.62
131	ILE	D	611	21.82	0.38	22.19	611		60.73	45.63	0.73
135	SER	D	615	0.15	0.00	0.16	615		3.72	0.17	0.00
136	GLU	D	616	13.36	29.94	43.30	616	HS	79.85	39.49	-0.11
139	ILE	D	619	23.60	3.67	27.28	619		86.49	84.20	0.89
140	ARG	D	620	11.12	24.75	35.88	620	HS	143.73	76.81	-0.18
141	HIS	D	621	1.96	2.26	4.22	621		86.84	13.46	0.13
142	SER	D	622	2.00	7.07	9.07	622	H	16.27	15.77	-0.18
143	PRO	D	623	7.17	15.36	22.53	623		83.29	20.45	-0.08
144	CYS	D	624	4.77	3.90	8.67	624		26.34	18.80	0.17
145	ASP	D	625	17.11	49.82	66.92	625	HS	80.07	80.07	-0.26
146	VAL	D	626	4.64	16.61	21.25	626	H	42.73	41.11	-0.25
147	LEU	D	627	53.06	0.86	53.92	627		78.25	69.22	1.11
148	VAL	D	628	40.46	11.20	51.66	628	H	67.39	62.33	0.56

Continued

Complex C-D																										Continued
Structure1													Structure2													
			POPSCOMP				PISA									POPSCOMP				PISA						
##	Res	Chain	ResNr	Phob	Phil	Tot	ResNr	HSDC	ASA	BSA	Δ G		##	Res	Chain	ResNr	Phob	Phil	Tot	ResNr	HSDC	ASA	BSA	Δ G		
													149	VAL	D	629	4.44	0.49	4.92	629		3.52	3.52	0.06		
													150	ARG	D	630	36.47	20.50	56.96	630	H	129.72	71.14	-0.79		
													151	ASN	D	631	0.76	8.72	9.48	631		44.35	11.32	-0.17		
													159	GLU	D	639	0.00	2.13	2.14	639		114.73	6.14	-0.10		

Complex A-C																							
Structure1												Structure2											
			POPSCOMP				PISA								POPSCOMP				PISA				
##	Res	Chain	ResNr	Phob	Phil	Tot	ResNr	HSDC	ASA	BSA	Δ G	##	Res	Chain	ResNr	Phob	Phil	Tot	ResNr	HSDC	ASA	BSA	Δ G
20	GLY	A	20	1.55	0.83	2.36	20		36.16	4.26	0.06	19	ASP	C	339	0.05	0.00	0.05					
48	ILE	A	48	1.98	0.00	1.99	48		29.38	6.03	0.10	20	GLY	C	340	2.58	0.76	3.35	340		36.85	6.83	0.04
49	ASP	A	49	2.20	4.79	6.97	49	HS	51.91	18.46	-0.14	46	HIS	C	366	5.51	4.36	9.87	366		25.20	17.22	0.52
51	ARG	A	51	21.10	58.75	79.85	51	HS	196.86	98.95	-0.77	47	VAL	C	367	0.01	0.44	0.45	367		18.23	0.61	-0.01
52	ALA	A	52	11.03	0.30	11.33	52		53.41	16.06	0.26	48	ILE	C	368	9.21	0.00	9.21	368		28.04	13.72	0.22
53	PHE	A	53	93.15	3.50	96.64	53		200.56	124.67	1.64	49	ASP	C	369	2.97	3.85	6.83	369	HS	43.57	22.33	0.16
54	SER	A	54	5.90	2.17	8.08	54		61.26	16.49	0.19	51	ARG	C	371	23.59	62.06	85.66	371	HS	194.18	95.45	-0.97
55	SER	A	55	1.34	0.26	1.60	55		125.77	7.04	0.02	52	ALA	C	372	8.64	0.22	8.87	372		46.89	13.56	0.22
56	VAL	A	56	6.87	0.12	6.98	56		62.74	29.99	0.47	53	PHE	C	373	79.66	5.21	84.87	373		199.98	110.24	1.37
58	ASN	A	58	0.03	0.21	0.24						54	SER	C	374	5.45	4.62	10.07	374		56.63	16.06	0.00
59	TYR	A	59	84.76	15.65	100.40	59		165.43	95.42	1.33	55	SER	C	375	12.78	5.98	18.76	375		106.76	14.85	0.11
60	ASP	A	60	3.53	8.70	12.23	60		39.93	11.51	-0.01	56	VAL	C	376	17.85	2.58	20.43	376		89.42	21.63	0.25
62	SER	A	62	7.19	12.94	20.14	62		56.79	23.48	0.02	58	ASN	C	378	0.62	1.42	2.05	378		108.70	1.27	0.01
63	MET	A	63	69.13	0.25	69.38	63		113.97	92.14	2.60	59	TYR	C	379	68.73	6.22	74.95	379		178.46	82.95	1.20
66	LYS	A	66	44.72	30.03	74.75	66		141.27	82.59	-0.55	60	ASP	C	380	5.04	6.50	11.53	380		27.20	8.55	0.12
67	ALA	A	67	8.46	0.66	9.12	67		28.73	24.00	0.38	62	SER	C	382	10.99	13.62	24.61	382		50.05	28.55	0.11
70	TYR	A	70	41.91	7.23	49.14	70		129.52	38.50	0.60	63	MET	C	383	63.20	0.92	64.13	383		114.90	102.44	2.72
71	ALA	A	71	0.00	0.01	0.01						66	LYS	C	386	12.68	16.81	29.49	386		132.37	41.99	-0.30
98	SER	A	98	1.89	0.47	2.36	98		40.28	10.91	0.15	67	ALA	C	387	13.14	1.21	14.35	387		31.44	28.30	0.41
99	PRO	A	99	0.18	0.00	0.17	99		40.53	0.84	0.01	68	THR	C	388	0.03	0.07	0.12					
100	LYS	A	100	3.66	10.93	14.59	100	H	67.53	27.43	-0.99	70	TYR	C	390	17.57	0.22	17.79	390		122.89	23.22	0.37
101	THR	A	101	16.34	1.50	17.83	101		64.91	33.33	0.52	71	ALA	C	391	0.93	0.08	1.00	391		1.26	0.17	0.00
104	THR	A	104	2.72	0.00	2.73	104		26.50	10.88	0.17	74	LEU	C	394	0.16	0.00	0.16					
105	LYS	A	105	12.98	10.32	23.30	105		130.97	22.72	-0.10	98	SER	C	418	0.87	5.24	6.11	418		37.27	12.64	-0.14
123	LEU	A	123	5.50	0.00	5.50	123		123.16	12.04	0.19	99	PRO	C	419	0.78	0.00	0.77	419		37.48	2.15	0.03
124	ASN	A	124	16.64	40.90	57.55	124	H	143.75	76.52	-0.48	100	LYS	C	420	10.54	20.57	31.11	420		80.88	52.49	-1.20
125	ALA	A	125	4.83	0.30	5.13	125		22.72	16.23	0.26	101	THR	C	421	13.81	1.72	15.52	421		59.47	38.47	0.58
128	ARG	A	128	41.94	54.78	96.72	128		214.40	99.72	-0.22	102	ALA	C	422	0.00	0.01	0.01					
129	LEU	A	129	42.80	0.08	42.88	129		90.54	59.30	0.95	104	THR	C	424	0.80	0.00	0.80	424		23.68	6.70	0.11
131	ILE	A	131	0.09	0.00	0.08	131		50.43	0.83	0.01	105	LYS	C	425	8.45	0.92	9.38	425		149.62	18.58	0.30
132	GLY	A	132	0.14	0.00	0.14						124	ASN	C	444	8.17	33.13	41.31	444		134.80	59.79	-0.44
133	SER	A	133	8.37	3.45	11.81	133		63.41	26.26	0.39	125	ALA	C	445	0.51	0.00	0.51	445		22.99	2.51	0.04
136	GLU	A	136	0.29	6.49	6.79	136		54.09	20.54	-0.29	128	ARG	C	448	35.43	60.07	95.50	448		210.74	108.88	-0.41
137	TYR	A	137	9.26	7.66	16.92	137		53.89	44.21	0.27	129	LEU	C	449	39.50	0.06	39.56	449		89.66	58.16	0.93
140	ARG	A	140	1.39	1.23	2.62	140		158.54	10.08	-0.13	133	SER	C	453	14.78	19.47	34.25	453	H	83.88	42.05	0.01
141	HIS	A	141	4.95	2.83	7.78	141		86.38	19.89	0.05	136	GLU	C	456	0.23	2.00	2.24	456		64.52	9.57	-0.11
												137	TYR	C	457	9.71	8.03	17.74	457		54.57	42.94	0.09
												140	ARG	C	460	0.69	8.93	9.61	460		176.44	19.55	-0.63
												141	HIS	C	461	4.23	4.03	8.27	461		91.70	21.24	-0.36

Complex B-D

Structure1

			POPSCOMP				PISA				
##	Res	Chain	ResNr	Phob	Phil	Tot	ResNr	HSDC	ASA	BSA	Δ G
20	GLY	B	180	1.67	0.48	2.14	180		43.69	5.18	0.08
48	ILE	B	208	8.06	0.00	8.06	208		36.58	14.23	0.23
49	ASP	B	209	0.10	1.76	1.86	209		68.28	10.06	-0.17
51	ARG	B	211	48.32	53.33	101.65	211	H	192.36	76.25	0.22
52	ALA	B	212	10.09	0.31	10.40	212		52.85	17.73	0.28
53	PHE	B	213	88.25	5.86	94.11	213		213.04	119.10	1.42
54	SER	B	214	4.53	0.88	5.40	214		45.03	8.70	0.14
55	SER	B	215	13.55	5.27	18.82	215		116.30	22.39	0.23
56	VAL	B	216	13.70	0.38	14.08	216		77.53	27.46	0.44
58	ASN	B	218	0.07	2.03	2.09	218		111.58	5.09	-0.05
59	TYR	B	219	49.88	23.13	73.01	219	H	112.24	109.09	1.09
60	ASP	B	220	0.16	0.34	0.50	220		20.28	0.12	0.00
62	SER	B	222	6.76	14.01	20.78	222		66.48	22.90	-0.03
63	MET	B	223	30.94	0.18	31.12	223		76.21	63.61	1.71
66	LYS	B	226	29.34	13.70	43.03	226	H	140.82	49.64	-0.04
67	ALA	B	227	10.45	0.77	11.22	227		28.61	24.83	0.38
70	TYR	B	230	33.06	6.85	39.92	230		124.73	38.00	0.60
71	ALA	B	231	0.00	0.02	0.02					
74	LEU	B	234	0.27	0.00	0.27					
98	SER	B	258	0.73	0.16	0.89	258		28.18	6.44	0.10
100	LYS	B	260	10.35	23.39	33.74	260		79.25	55.00	-1.16
101	THR	B	261	16.25	5.62	21.87	261		58.50	30.81	0.24
104	THR	B	264	3.55	0.17	3.72	264		23.52	11.14	0.18
123	LEU	B	283	9.27	0.34	9.61	283		132.25	20.17	0.32
124	ASN	B	284	25.28	64.33	89.60	284	H	152.80	84.88	-0.75
125	ALA	B	285	3.42	0.34	3.77	285		24.53	8.18	0.13
128	ARG	B	288	51.53	96.31	147.84	288	H	220.71	123.27	-1.35
129	LEU	B	289	10.40	0.06	10.46	289		66.82	33.37	0.53
133	SER	B	293	12.15	4.45	16.59	293		83.86	31.51	0.47
136	GLU	B	296	8.54	35.77	44.32	296		102.26	41.18	-0.49
137	TYR	B	297	7.44	6.21	13.66	297	H	47.22	34.93	0.06
140	ARG	B	300	0.69	1.70	2.39	300		181.13	4.37	-0.05
141	HIS	B	301	4.94	3.08	8.02	301		69.94	23.12	-0.14

Structure2

			POPSCOMP				PISA				
##	Res	Chain	ResNr	Phob	Phil	Tot	ResNr	HSDC	ASA	BSA	Δ G
19	ASP	D	499	0.00	0.48	0.48	499		69.42	2.46	-0.03
20	GLY	D	500	4.40	2.38	6.79	500		17.61	8.10	0.06
26	HIS	D	526	0.31	0.00	0.31					
49	ASP	D	529	0.14	1.62	1.77	529		65.95	4.30	-0.07
51	ARG	D	531	19.93	30.59	50.52	531	H	162.06	62.78	-0.20
52	ALA	D	532	10.71	0.35	11.07	532		54.13	17.60	0.28
53	PHE	D	533	78.24	8.34	86.58	533		200.39	123.86	1.44
54	SER	D	534	5.62	2.17	7.79	534		50.12	14.17	0.17
55	SER	D	535	19.74	16.82	36.57	535	H	115.26	44.27	0.11
56	VAL	D	536	3.42	0.31	3.72	536		63.78	15.74	0.25
58	ASN	D	538	0.03	0.14	0.17					
59	TYR	D	539	49.77	7.06	56.83	539		162.24	58.51	0.87
60	ASP	D	540	6.62	10.56	17.19	540	H	32.33	11.21	0.02
61	THR	D	541	0.00	0.06	0.07	541				
62	SER	D	542	4.88	5.54	10.42	542		60.96	14.18	-0.10
63	MET	D	543	63.70	1.21	64.90	543		98.38	87.48	2.52
64	ALA	D	544	0.00	0.01	0.02					
66	LYS	D	546	18.67	0.15	18.82	546		121.18	46.03	0.74
67	ALA	D	547	5.99	0.39	6.37	547		34.74	21.42	0.34
70	TYR	D	550	39.78	9.61	49.38	550		124.50	34.12	0.50
98	SER	D	578	1.87	3.98	5.85	578		13.90	9.94	-0.06
100	LYS	D	580	2.06	0.61	2.68	580		60.47	14.32	0.20
101	THR	D	581	14.96	3.09	18.05	581	H	54.09	39.27	0.56
102	ALA	D	582	0.05	0.11	0.17					
104	THR	D	584	4.11	0.46	4.58	584		23.89	11.49	0.18
105	LYS	D	585	5.62	0.08	5.70	585		121.79	12.72	0.20
123	LEU	D	603	20.74	6.55	27.30	603	H	112.13	46.44	0.31
124	ASN	D	604	21.52	41.17	62.69	604		152.41	66.72	-0.31
125	ALA	D	605	4.20	0.69	4.89	605		16.36	13.21	0.21
126	VAL	D	606	0.04	0.00	0.03					
128	ARG	D	608	35.15	62.35	97.50	608	H	205.64	90.35	-0.38
129	LEU	D	609	40.49	0.04	40.52	609		91.83	64.51	1.03
132	GLY	D	612	0.18	0.00	0.18	612		7.18	0.17	0.00
133	SER	D	613	7.82	15.95	23.77	613		79.01	40.67	-0.15
136	GLU	D	616	5.82	22.98	28.81	616		79.85	32.35	-0.33
137	TYR	D	617	5.61	5.17	10.77	617	H	42.10	35.88	0.19
140	ARG	D	620	0.09	3.17	3.26	620		143.73	4.81	-0.14
141	HIS	D	621	4.00	4.16	8.16	621		86.84	25.94	-0.49

Complex A-D																							
Structure1												Structure2											
			POPSCOMP				PISA								POPSCOMP				PISA				
##	Res	Chain	ResNr	Phob	Phil	Tot	ResNr	HSDC	ASA	BSA	Δ G	##	Res	Chain	ResNr	Phob	Phil	Tot	ResNr	HSDC	ASA	BSA	Δ G
140	ARG	A	140	0.37	3.03	3.4	140		158.54	17.67	-0.39	136	GLU	D	616	0	0.68	0.68	616		79.85	2.05	-0.02
												140	ARG	D	620	0.09	2.58	2.68	620		143.73	15.27	-0.44

Complex B-C																							
Structure1												Structure2											
			POPSCOMP				PISA								POPSCOMP				PISA				
##	Res	Chain	ResNr	Phob	Phil	Tot	ResNr	HSDC	ASA	BSA	Δ G	##	Res	Chain	ResNr	Phob	Phil	Tot	ResNr	HSDC	ASA	BSA	Δ G
136	GLU	B	296	5.61	20.79	26.41	136	HS	102.26	27.31	-0.26	136	GLU	C	456	0.13	2.47	2.6	456		64.52	0.61	-0.01
140	ARG	B	300	6.12	15.36	21.48	140		181.13	43.92	-0.75	140	ARG	C	460	3.84	33.43	37.28	460	HS	176.44	60.06	-1.33
												141	HIS	C	461	1.11	1.13	2.26	461		91.70	16.11	-0.30
